# A Hydrothermal-Sedimentary Context for the Origin of Life

**DOI:** 10.1089/ast.2017.1680

**Published:** 2018-03-01

**Authors:** F. Westall, K. Hickman-Lewis, N. Hinman, P. Gautret, K.A. Campbell, J.G. Bréhéret, F. Foucher, A. Hubert, S. Sorieul, A.V. Dass, T.P. Kee, T. Georgelin, A. Brack

**Affiliations:** ^1^ CNRS-Centre de Biophysique Moléculaire, Orléans, France.; ^2^ Dipartmento di Scienze biologiche, geologiche e ambientale, Università di Bologna, Bologna, Italy.; ^3^ Geosciences, University of Montana, Missoula, Montana, USA.; ^4^ University of Orléans, ISTO, UMR 7327, Orléans, France, and CNRS, ISTO, UMR 7327, Orléans, France, and BRGM, ISTO, UMR 7327, Orléans, France.; ^5^ School of Environment, The University of Auckland, Auckland, New Zealand.; ^6^ GéoHydrosytèmes Continentaux, Faculté des Sciences et Techniques, Université François-Rabelais de Tours, Tours, France.; ^7^ University of Bordeaux, CNRS, IN2P3, CENBG, UMR5797, Gradignan, France.; ^8^ School of Chemistry, University of Leeds, Leeds, UK.; ^9^ Sorbonne Universités, UPMC Paris 06, CNRS UMR 7197, Laboratoire de Réactivité de Surface, Paris, France.

## Abstract

Critical to the origin of life are the ingredients of life, of course, but also the physical and chemical conditions in which prebiotic chemical reactions can take place. These factors place constraints on the types of Hadean environment in which life could have emerged. Many locations, ranging from hydrothermal vents and pumice rafts, through volcanic-hosted splash pools to continental springs and rivers, have been proposed for the emergence of life on Earth, each with respective advantages and certain disadvantages. However, there is another, hitherto unrecognized environment that, on the Hadean Earth (4.5–4.0 Ga), would have been more important than any other in terms of spatial and temporal scale: the sedimentary layer between oceanic crust and seawater. Using as an example sediments from the 3.5–3.33 Ga Barberton Greenstone Belt, South Africa, analogous at least on a local scale to those of the Hadean eon, we document constant permeation of the porous, carbonaceous, and reactive sedimentary layer by hydrothermal fluids emanating from the crust. This partially UV-protected, subaqueous sedimentary environment, characterized by physical and chemical gradients, represented a widespread system of miniature chemical reactors in which the production and complexification of prebiotic molecules could have led to the origin of life. Key Words: Origin of life—Hadean environment—Mineral surface reactions—Hydrothermal fluids—Archean volcanic sediments. Astrobiology 18, 259–293.

## 1. Introduction

In this section, we briefly overview the basic requirements for prebiotic chemistry and the emergence of life, underlining the importance of the mineral world, in particular for the former. Considering the significance of the geological environment for the origin of life, we outline the present understanding regarding environmental conditions reigning on early Earth. We then consider various previously proposed environments in which life might have arisen. With this background, the objective of our study is to show, using an examination of Paleoarchean volcanic sediments strongly influenced by hydrothermal fluids as a benchmark, that volcanic sediments could have played an important role in prebiotic chemistry and in the increasing complexity of prebiotic chemical systems eventually leading to the origin of life.

### 1.1. Requirements for prebiotic chemistry and the emergence of life

When considering the local environment (or environments) in which life could have emerged, the prebiotic processes that led to the origin of cellular life and the physical and chemical conditions in which these processes could occur need to be taken into account. There are three critical steps that lead toward cell formation (Table 1): (1) concentration of the molecular components that participate in prebiotic reactions and control of water activity, (2) stabilization and structural conformation of molecules, and (3) chemical evolution through complexification. Experimental data show that the aforementioned processes are greatly aided by the presence of mineral surfaces, as summarized by Hazen and Sverjensky (2010) and Dass *et al.* (2016), thus underlining the importance of the mineral world for prebiotic chemistry and the emergence of life.

**Table 1.** Important Processes for the Emergence of Life

*Process*	*Rationale*	*Means*
Concentration of prebiotic components	Favors probability of interactions between molecules	Confinement within small compartments (pores), voids and interstitial spaces on mineral surfaces, chelation to mineral surfaces, including silica gel, etc.
Conformation, structuration, and stabilization	Molecular orientation affects reactions between molecules (*e.g.,* stereochemistry); prevention of molecular degradation (*e.g.,* of ribose); permits reactivity	*e.g.,* chelation on mineral surfaces
Complexification	Buildup and polymerization of macromolecules necessary for prebiotic processes and emergence of life	*e.g.,* through self-assembly, vesicle formation, systems chemistry

*(1) Concentration:* Concentration of essential biomolecules is of prime importance in understanding the origins of life. The effectiveness of interfaces between minerals and aqueous solutions in this regard was highlighted by Hazen and Sverjensky (2010). Several studies (Hazen, 2001, 2004; Cleaves *et al.,* 2012; Grosch and Hazen, 2015) have emphasized the importance of crystal surfaces and how they provide an effective substrate upon which the concentration and catalysis of prebiotic molecules could be possible. Adsorption is of critical importance in assisting concentration. Concentration and “crowding” of molecules is essential in order to promote reactions between the molecules that could lead to coevolution of their reaction mechanisms (Copley *et al.,* 2007). Spitzer and Poolman (2009) underlined the importance of “micro-spaces” for confinement “that select[s] (create, import, retain, or expel) protobiomolecules.” Various types of microspaces have been suggested, such as iron sulfide compartments in hydrothermal vents (Russell and Hall, 1997), porous “beehive-like” hydrothermal structures (such as those described by Tivey and Delaney, 1986), pores at the surfaces of minerals (Hazen and Sverjensky, 2010), crevices within minerals (*e.g.,* pumice; Brasier *et al.,* 2011, 2013), among others. Certain mineral surfaces and/or intracrystalline spaces can also concentrate organic molecules, such as clays (Reid and Orgel, 1967), zeolites (Smith *et al.,* 1999), and feldspars (Parsons *et al.,* 1998). Spitzer and Poolman (2009) suggested pore sizes of 0.1–1.0 μm as being of relevant size for molecular concentration. In addition, the recognition that contemporary biological cells retain, concentrate, and manipulate biomolecules within an environment that is inherently a hydrogel (Pollack, 2001; Trevors and Pollack, 2005; Trevors, 2010, 2011a, 2011b; Saha *et al.,* 2014) has led to suggestions that geologically relevant gel environments in which molecular crowding could take place would also represent an environment of prebiotic microspace.

*(2) Stabilization and structural conformation:* Molecular stabilization plays an important role in prebiotic chemistry. Given the short lifetimes of biomolecules, such as ribose, it is hard to envisage the reactivity of such fragile molecules in the harsh conditions at the surface of early Earth. Minerals could potentially favor molecular stabilization, prolong their lifetimes, and thus prevent their rapid degradation, for example, the stabilization of ribose on minerals (Georgelin *et al.,* 2013, Akouche *et al.,* 2016, and Lambert *et al.,* 2010, on silicates, or Ricardo *et al.,* 2004, on borates). Structural conformation and orientation of molecules permits reactivity and selectivity of organic reactions. Brack and Orgel (1975) showed that the addition of salts, for instance 0.1 *M* NaCl, spontaneously produces an asymmetrical beta-sheet bilayer of peptides (with a hydrophobic interior and a hydrophilic exterior) (note, for comparison, that the concentration of NaCl in modern seawater is 0.6 *M*). Bertrand and Brack (2000) demonstrated catalysis of beta-sheet formation on cadmium sulfide. Jonsson *et al.* (2009) experimentally showed that l-glutamate could be adsorbed onto the surface of rutile in salt solutions and that the role of orientation in adsorption could potentially affect chemical reactivity.

*(3) Chemical evolution:* Studies in systems chemistry at the molecular level support indications of the progressive buildup of molecular complexity (Ruiz-Mirazo *et al.,* 2014; Islam and Powner, 2017). Dass *et al.* (2016) emphasized the relevance of minerals in stochastic systems chemistry. Patel *et al.* (2015), using a systems chemistry approach, suggested that the precursors of ribonucleotides, amino acids, and lipids originated simultaneously from a single source. Clearly, we still do not understand the fundamental mechanisms by which the first forms of biological life emerged. However, it is recognized that such emergence resulted from energy being transduced from one form to another and for some of that energy to have been used to drive spontaneous self-organization, or complexity. In this context, “complexity” refers to the mutual and integrated communication between chemical species rather than the variety of chemical species present *per se*. It is only through this connectivity that self-organization is revealed. While we recognize the fundamental importance of this concept to the emergence of life, a detailed discussion of biosynthesis is beyond the scope of this paper. The interested reader is encouraged to read excellent contributions on this subject by, for example, Martin and Russell (2003) and Russell *et al.* (2010).

The above, briefly described processes that led to the emergence of life were controlled by physicochemical conditions of the environment, including element availability, water temperature, pH, ionic strength, energy, irradiation, gradients, and molecular diffusion (Table 2). C, H, N, O, and P are the basic ingredients for prebiotic chemistry, as elements with minor endogenic and major exogenic origins (Pizzarello and Shock, 2010; Dass *et al.,* 2016). Other elements, such as S and transition metals, become important for the emergence of cellular life and primitive metabolisms. Temperature, in addition to concentration and pressure, controls the kinetics of the many reactions that occur in prebiotic chemistry: it facilitates the increased probability of intermolecular collision and helps overcome energy barriers. Higher temperatures also result in dehydration leading to the condensation (concentration) of molecules, mainly on mineral surfaces. However, temperature is limiting to cellular life. Above ∼120°C, molecular bonds break, such as RNA/DNA nucleotides (Kashefi and Lovley, 2003). Molecular reactions in aqueous solution are linked to the pH of the solution, with different pH values favoring different kinds of reactions, thus permitting the emergence of a wide range of organic molecules (*e.g.,* Gull *et al.,* 2015). Reactions requiring sugars, for instance, require an alkaline pH, while many others can take place at acidic pH values. Ionic strength is an important factor for two reasons. In solution, the nature and the activity of salts can stabilize and affect structural orientation of organic species and polymers, leading to spontaneous molecular assembly, for example, the formation of surfactant micelles (Spitzer and Poolman, 2009). It also influences the adsorption of organic molecules onto mineral surfaces; for example, an increase of ionic strength overcomes electrostatic interactions, thus controlling adsorption. This contributes to the selection of organic molecules that participate in the emergence of cellular life (Spitzer and Poolman, 2009). Ionic strength can be controlled by the nature of mineral and chemical functions available at the surface of the mineral.

**Table 2.** The Physicochemical Environment of the Origin of Life

	*Prebiotic chemistry*		*Emergence of life*	
*Parameter*	*What*	*How or where*	*What*	*How or where*
**Element availability**	CHNOPS essential for life	From endogenous and exogenous CHNOPS molecules	Chemical bonding of these elements—CHNO—range in covalency, allowing sufficiently strong yet penetrable bonding among these elements. S and P are also penetrable but not involved in long chain formation. Assembly of atoms into discrete molecules is also a form of molecular complexification. Mutual cooperative interactions between molecules so that self-organization can emerge and function can be expressed.	By enabling stable linear carbon chains while introducing elements with different oxidation-reduction and pH reactivities that allow reactions without destruction (enzymes) or formation of metastable intermediates leading, ultimately, to sustainable, stable products. Within environments which presented free energy, molecular building blocks and conducive gradients of physicochemical properties so as to encourage mutually cooperative molecular interactions in localized spaces.
**Temperature**	Increases the frequency with which energetic molecular collisions can occur; helps pass activation energy barrier	Probability of intermolecular collision is increased, activation energy supplied by thermal means to cross the energy barrier (heat in hydrothermal systems), helps in dehydration for condensations by removal of water	High temperatures will break up molecular bonds (DNA/RNA nucleotides are stable up to 120°C)	Mixture of high *T* hydrothermal fluids with lower *T* seawater varies temperature between the two end members according to flux—see gradients, but also advective flux
**pH**	Different chemical processes occur at different pH values	Alkaline-acid hydrothermal fluids; acidic seawater; mixtures of hydrothermal and seawater; *e.g.,* amino acid coupling to produce peptides at high pH and general acid-catalyzed hydrolysis processes at low pH.	Variable pH changes structure and surface properties of colloids, sols, and polymers. Also allows nucleophilic reactions (substitutions), condensation reactions.	Variable pH for protocells, by allowing reversible and irreversible reactions involving CHNO (S, P) components
**Ionic strength**	Helps stabilization and structural organization of organic molecules, spontaneous self-assembly phenomena; gradients are important for physicochemical properties	Complexification processes, *e.g.,* self-assembly, form a concentration of monomers through the formation of surfactant micelles, interfacial adsorption of ionic surfactants and formation of aqueous colloidal crystals	Salts necessary for protocells, but minimum water activity is 0.5	By changing the surface charge of particles and by changing the properties and stability of micelles. Seawater, hydrothermal, pore water fluids.
**Energy**	Energy is necessary for fueling reactions and primitive metabolisms	*e.g.,* ionizing radiation for prebiotic reactions; energy from exothermic reactions; heat from hydrothermal systems	For primitive metabolism	Assuming that there were, originally, only reduced compounds with various oxidation states (inorganic components) and that organic components delivered to Earth were in variable oxidation states. Oxidation of reduced inorganic compounds, *e.g.,* NH_4_, NO_2_, S_2_, S_0_, H_2_ and Fe^2+^; oxidation of organic compounds produces phosphorylated molecules.
**Radiation**	Useful for some prebiotic reactions but mainly destructive for organic molecules	UV, radiogenic species (U, Th, etc.)	Destructive for biomolecules	By introducing random flaws in the structure of organic molecules. By introducing temporary elevated energy states in inorganic components and transition elements that enable potential bonding with organic components. UV, radiogenic species (U, Th, etc.).
**Gradients**	A means of maintaining systems out of equilibrium	Dissipation of energy. Sinks for this dissipated energy are potential reaction sites …	Necessary for maintaining far-from-equilibrium systems; to gain and dissipate energy, reactants, and products	*e.g.,* temperature, pH, ionic gradients
**Molecular diffusion**	Diffusion of molecules into and out of the above-mentioned compartments to permit molecular reactions	In solvents, *e.g.,* hydrothermal fluids, seawater, pore waters, mixtures	Diffusion of molecules into and out of the above-mentioned compartments to permit molecular reactions	In solvents, *e.g.,* hydrothermal fluids, seawater, pore waters, mixtures

All reactions require energy. Ionizing radiation is an important source of energy for prebiotic chemistry. Its ubiquity, energy introduction method, and the effectiveness of its reactions via free radicals make it very efficient for prebiotic chemistry, both in frozen ices (for extraterrestrial processes) and in water (for processes occurring on early Earth) (Negrón-Mendoza *et al.,* 2016). For example, cosmic radiation at various wavelengths has been invoked as an extraterrestrial cause of enantiomeric excesses of organic molecules in carbonaceous chondrites (Bonner, 1991; Bailey, 2001). Meinert *et al.* (2016) reported obtaining ribose and other sugars from UV irradiation of interstellar ice analogues. Other potential sources of radiation could have come from ^40^K, which would have been relatively enriched in Hadean granitoids. However, this would require weathering and exposure on the relatively rare Hadean landmasses. Other sources of energy include exothermic reactions or heat from hydrothermal systems. Energy for primitive cellular metabolisms (chemotrophic) was provided by the oxidation of reduced inorganic compounds (*e.g.,* NH_4_, NO_2_, SO_3_, SO, H_2_, and Fe^(II)^), as well as the oxidation of organic compounds, reactions that all produce the phosphorylated, energy storage molecule adenosine triphosphate (ATP).

The radiation environment can have both positive and negative consequences. While radiation serves as an energy source for certain prebiotic reactions, it is generally deleterious to both molecular complexification and cellular life, inducing molecular breakdown. Note, however, that certain present-day extremophilic microorganisms are able to survive high doses of radiation (*Deinococcus radiodurans* survives up to 5000 Gy of radiation; Cox and Battista, 2005) and that early life was clearly able to withstand the relatively high radiation environment of early Earth (Westall *et al.,* 2006a).

Gradients (*e.g.,* of temperature, pH, ionic strength, energy) are a means of maintaining systems out of equilibrium. They aid dissipation of energy, and sinks of dissipated energy are potential reaction sites (Dass *et al.,* 2016). Gradients are also important for the functioning of protocells, again mainly for energy dissipation. Of prime importance is also the diffusion of essential components for prebiotic chemistry and primitive metabolisms, transported in hydrothermal fluids, seawater, pore waters (in porous materials), and mixtures of these into and out of compartments (gels, vesicles, pores in rocks and minerals) in which prebiotic chemistry could take place.

### 1.2. Early Earth environment

As the context for prebiotic chemistry and the emergence of life, in this section we review what is known or surmised of the environment of early Earth (see also reviews by Russell and Arndt, 2005, and Kamber, 2015). For this we need to keep in mind that, given the lack of preserved Hadean (4.5–3.85 Ga, *sensu* Bleeker, 2004; Kamber 2015) crust, our understanding of the Hadean environment is extremely sketchy. Moreover, there will be a difference between the global-scale situation and the microscopic- to local-scale environments in which prebiotic chemistry occurred and protocells emerged. The lack of Hadean crust has been attributed to destruction and resurfacing during the period of the hypothesized Late Heavy Bombardment, 4.1–3.85 Ga (Bottke *et al.,* 2012), when the inner planets were subjected to intense and deleterious bombardment by extraterrestrial bodies (Kemp *et al.,* 2010; Marchi *et al.,* 2014), and to catastrophic recycling of the early crust, possibly combining collapse of the single lithospheric plate that covered the planet (stagnant lid, after Griffin *et al.,* 2014) with episodic, short-lived plate tectonics (Debaille *et al.,* 2013). However, the Late Heavy Bombardment model has since been revisited to imply that the impact “spike” resembled not catastrophic bombardment but rather a long-drawn-out period of more intense bombardment throughout the Hadean (Zellner, 2017). Note that, in a recent reevaluation of the models pertaining to asteroid bombardment during the Hadean, Sleep (2016) estimated that the impacts would have been relatively benign and there would not have been more than one impact severe enough to affect early life-forms.

Despite the lack of crustal preservation, some information about the Hadean can be deduced from modeling (*e.g.,* Johnson *et al.,* 2014), from proxies such as the characteristics of zircon crystals eroded from buried Hadean crust and recycled into younger sedimentary formations (*e.g.,* Mojzsis *et al.,* 2001; Wilde *et al.,* 2001; Nemchin *et al.,* 2006), and from remnant geochemical signatures in the preserved Eoarchean-Paleoarchean cratons (*e.g.,* Kamber *et al.,* 2003). (Note that carbon with a δ^13^C signature of −24‰ was detected in inclusions in one Hadean zircon crystal [Bell *et al.,* 2015], a value that could be either biogenic or abiogenic.)

The kind of global-scale information relevant to prebiotic chemistry concerns the composition of the crust (*i.e.,* mineral availability); the presence of water, the distribution of emergent land masses; temperatures; indications of additional physicochemical characteristics of the water (temperature, pH, ionic strength); and additional parameters such as gradients, diffusion, and energy sources for prebiotic reactions and cellular metabolism.

From geochemical analyses of Eoarchean (3.85–3.5 Ga) cratons underlain by Hadean crust, it appears that the Hadean crust had a mafic signature (Kamber *et al.,* 2003). Thus, typical minerals available would have included the ferromagnesian minerals olivine and pyroxene, as well as plagioclase and calcic feldspars and the iron oxide, magnetite. The products of aqueous and hydrothermal alteration of these minerals would have been varied, including phyllosilicates (montmorillonite, smectites, and zeolites) and Fe and Ti oxides. Associated hydrothermal minerals could have included mainly Fe (with more minor Ca and Mg) carbonates, zeolites, ferrous and ferric oxyhydroxides, various sulfides (*e.g.,* Fe, Ni, Zn), and amorphous silica (gels, cherts).

Evidence for water on the Hadean Earth comes from mineralogical and geochemical sources. Zircon crystals, formed during remelting of buried, hydrated Hadean crust, were eroded from subsequently exposed crust and were deposited as inherited detrital particles in younger, Mesoarchean (3.1–2.85 Ga) sediments (*e.g.,* Mojzsis *et al.,* 2001; Wilde *et al.,* 2001). The existence of detrital Hadean zircons suggests that liquid water was present at the surface of the Hadean Earth, possibly already by 4.36 Ga, the age of the oldest zircons (Mojzsis *et al.,* 2001; Wilde *et al.,* 2001; Nemchin *et al.,* 2006). Kramers (2003) similarly concluded that there was water on the Hadean Earth, based on a comparison of the abundances of light and gaseous elements (H, C, N, Ne, Ar, Cl, Br, Kr, I, and Xe) in the outer Earth reservoirs (atmosphere-hydrosphere, continental crust, and MORB-source mantle) with solar matter and carbonaceous chondrites. The temperature of water in the Hadean crust has been debated. An initial estimate of <150°C based on heavy δ^18^O isotopes by Wilde *et al.* (2001) in Hadean zircon crystals was demonstrated to be erroneous and, as indicated by Nemchin *et al.* (2006), due to later resetting or contamination. Hadean crustal temperatures would have governed hydrothermal circulation and the temperatures of hydrothermal fluids. Sleep (2000) and Zahnle *et al.* (2007) modeled relatively high heat flow rates in the early crust of from 160 up to 500 mW/m^2^, due to heating by the decay of radiogenic isotopes, such as U, Th, Al, and K. More recently, studies on inclusions in Hadean zircons suggest that they formed at relatively low temperatures and, therefore, heat flow in the Hadean crust was low, ∼40–80 mW/m^2^ (Hopkins *et al.,* 2010), that is, values as low as the lowest heat flow measured on the present-day Earth. For a recent review of the heat flow constraints and interactions of the hydrosphere/lithosphere with respect to the inset of plate tectonics, the interested reader is referred to the work of Korenaga (2013), who suggested that, on the basis of theoretical considerations and available observational constraints, plate tectonics driven by mantle heat flow could have initiated in the Hadean.

Bickle *et al.* (1994) concluded from thermal, tectonic, and isostatic constraints that, at least during the Archean (3.85 to ∼2.5 Ga), oceanic basins contained several kilometers of water but that the Archean continents were mostly submerged and characterized by shallow-water sediment sequences. The thin layers of sediments preserved from the Eoarchean indicate that, during that period, there was little exposed landmass. This seems also to have been the situation for the Hadean (Russell and Arndt, 2005).

Temperatures at the early Earth surface are highly debated, especially considering lower illumination from the “Faint Young Sun” (Sagan and Mullen, 1972). Various processes have been evoked to explain conditions conducive to the presence of liquid water on the Hadean/Archean Earth. For instance, Walker (1985) and Kasting (1993) modeled a thicker CO_2_ atmosphere. This is supported by mineralogical data, for example, the presence of siderite in equilibrium with magnetite in Archean shallow-marine sediments (Rosing *et al.,* 2010). It has been hypothesized that the early atmosphere contained certain amounts of “greenhouse” gases, such as methane (Pavlov *et al.,* 2000), or volcanically outgassed nitrogen (Mather *et al.,* 2004; Goldblatt *et al.,* 2006). However, at least with respect to CH_4_, a large amount of such gases in the atmosphere would have led to an organic haze, which would have prevented the penetration of sunlight to the surface and resulted in the freezing of Earth (Haqq-Misra *et al.,* 2008). Yet another hypothesis evokes a lower albedo than today for early Earth on the grounds that cloud nuclei today consist largely of oxidized sulfur species of biological (plant and algal, *i.e.,* eukaryotic) origin and the lack of such evolved organisms in the Hadean and the Archean would have hindered cloud formation (Rondanelli and Lindzen, 2010; Rosing *et al.,* 2010). Note that Sleep *et al.* (2008) proposed that the initial solar greenhouse might have been hot based on calculations related to tidal and internal heat flux.

Wide-ranging estimates of Paleoarchean (3.5–3.1 Ga) ocean temperatures made by using oxygen, silicon, and hydrogen isotopic signatures preserved in chert sediments dating back to 3.5 Ga concur that seawater was above freezing, at least at the sediment-seawater layer. Cooler temperatures down to ∼26°C were interpreted by Hren *et al.* (2009) and Blake *et al.* (2010), while warmer seawater temperatures (>50–60°C) have been estimated from the isotopic studies of van den Boorn *et al.* (2010), Marin-Carbonne *et al.* (2014), and Tartèse *et al.* (2017). The studies estimating higher temperatures note the influence of hydrothermal fluids on ambient seafloor temperatures.

Apart from the isotopic record, mineralogical and sedimentological data from the Paleoarchean also provide constraints on surface temperatures. The sediments in the Onverwacht Group of the Barberton Greenstone Belt document typical marine sedimentary deposition (Tice and Lowe, 2004; de Vries *et al.,* 2010; Westall *et al.,* 2015a). Some of the shallow-water sediments record tidal signatures in the form of oscillatory ripple marks (Westall *et al.,* 2015a), evaporite minerals (Lowe and Fisher Worrell, 1999), and desiccation cracks associated with evaporitic mineral suites (Westall *et al.,* 2006a), all indicating formation in a relatively warm environment, since the coastal regions would be the first to freeze. Further geological evidence comes from mineralogy with relatively high concentrations of atmospheric CO_2_, which supports temperatures warm enough to permit liquid water, as interpreted on the basis of the presence of siderite (Hessler *et al.,* 2004) and nahcolite (Tice and Lowe, 2006) in the Paleoarchean sediments. On one hand, it was recently suggested that the Paleoarchean Earth was glaciated, based on finely laminated sediments in the Paleoarchean Barberton Greenstone Belt that were interpreted to be glacial diamictites (de Wit and Furnes, 2016). However, the sediments described by de Wit and Furnes (2016) are very similar to other finely laminated sediments from the Onverwacht Group that exhibit clear indications of deposition in shallow, quiet water environments in at least warm seawater.

But what of temperatures during the Hadean? As noted above, the indication of hydrated, Hadean mafic crust (zircon formation) and light element abundances suggest that there was indeed water at the surface of the crust. However, Sleep *et al.* (2008) concluded that the critical surface temperatures permitting liquid water in a Hadean CO_2_ atmosphere could have been maintained for only up to 20 million years following the cooling of Earth after the Moon-forming impact. Their estimations, however, do not take into account the aforementioned greenhouse gases, such as methane (Pavlov *et al.,* 2000) or volcanically emitted nitrogen (Mather *et al.,* 2004; Goldblatt *et al.,* 2006). Zahnle *et al.* (2007) noted that removal of CO_2_ from the early atmosphere by subduction of precipitated carbonate could lead to a cold, frozen surface; but, if carbonate removal (subduction) were inefficient, CO_2_ partial pressures in the atmosphere would be sufficient to keep the surface warm enough to maintain liquid water. Indeed, the rheology of Hadean crust differed from that of later crust and may have been unable to support efficient modern-style plate tectonics (Stern, 2005, 2008). In fact, the earliest preserved evidence of modern tectonic cycling is at 3.2 Ga (Heubeck and Lowe, 1994; Stern, 2005; Van Kranendonk *et al.,* 2010). Removal of atmospheric CO_2_ by alteration of atmospheric dust caused by impacts was also invoked by Nisbet and Sleep (2001). Russell and Arndt (2005) suggested that atmospheric temperatures during the Hadean possibly oscillated between warm to hot and short-lived cold to freezing periods, with continuous eruption of volcanic CO_2_ into the atmosphere rapidly heating it up (*i.e.,* hysteresis).

It is clear that, owing to lack of geological evidence, the jury is out on the question of whether the surface of the Hadean Earth was frozen. Nevertheless, noting the discussion above about the geological evidence for liquid and warm to hot seawater temperatures (up to ∼60°C) by 3.5 Ga, the presence of more abundant radiogenic heat sources in the crust (U, Th, and K), and the fact that the crust was likely characterized by abundant hydrothermal activity (*cf.* Russell and Arndt, 2005), for the purposes of this study we assume that, during the Hadean, *seawater at the interface with the crust* must have been liquid, irrespective of whether deeper bodies of water were covered with ice at their surfaces.

The pH of the early oceans has also been a source of debate. The high partial pressure of CO_2_ in the Hadean-Paleoarchean atmosphere would have produced acid rain and concomitantly acidic seawater (Grotzinger and Kasting, 1993). This is supported through modeling by Morse and Mackenzie (1998), who estimated a pH ∼5.8 in the Hadean, gradually increasing to more neutral values in the Archean. On the other hand, Kempe and Degens (1985) proposed that the Hadean oceans were alkaline, owing to seawater alteration of the largely mafic/ultramafic ocean crust, which would have led to the deposition of significant amounts of carbonate. Circumneutral to alkaline pH values for the early oceans, similar to those of modern oceans, were also suggested by Friend *et al.* (2008) on the basis of trace elemental analyses of surviving Eoarchean rocks from the Isua Greenstone Belt in Greenland. Against this latter suggestion and in favor of acidic pH, the rock record from Early Archean sedimentary formations does not document large-scale carbonate deposits, although small amounts of carbonates did form from alteration of the mafic/ultramafic volcanics. Moreover, iron-containing formations are prevalent in the Paleoarchean rock formations, but Fe formation in open water cannot occur at pH 8 in a massive CO_2_ atmosphere, thus arguing for generally acidic conditions.

### 1.3. Environments in which life could have emerged

The requirements for prebiotic processes and very early cellular life (Tables 1 and 2), together with what may be surmised about Hadean surface environments, must be taken into account when considering a geological scenario for the emergence of life. We have subdivided proposed environments from previous literature as follows: (1) submarine hydrothermal vents, (2) floating pumice rafts, (3) subaerial geysers, and (4) volcanic-hosted splash pools. Their properties with respect to prebiotic processes and the emergence of life are summarized in Tables 3a–3d. Here, we briefly discuss each kind of environment and assess the advantages and shortcomings in their suitability as locations for life's emergence.

#### 1.3.1. Submarine hydrothermal vents

Submarine hydrothermal vents were first proposed as a possible location for the origin and early evolution of life by Baross and Hoffman (1985) on the grounds that physical and chemical gradients in hydrothermal systems “provided the necessary multiple pathways for the abiotic synthesis of chemical compounds, origin and evolution of ‘precells’ and ‘precell’ communities, and ultimately the evolution of free-living organisms.” Their hypothesis was further developed by Russell and Hall (1997), who proposed that the emergence of life within iron monosulfide bubbles (the mineral habit of which could produce “membranes” that mimic cell boundaries) formed at the gradient between hot (*e.g.,* up to 150°C) alkaline hydrothermal fluids and lower-temperature (but still hot, ∼90°C) acid seawater. The membranes would have catalyzed the synthesis of organic anions by hydrogenation and carboxylation of hydrothermal organic primers, resulting in the condensation of the organic molecules to polymers. Martin and Russell (2003) and Martin *et al.* (2008) furthered this scenario from the perspective of primitive metabolisms, drawing attention to the similarity between the H_2_-dependent chemistry of transition-metal sulfide catalysts and those catalysts and metabolisms involved in the CO_2_-reducing biochemistry of modern microorganisms. The exothermic synthesis of acetate and CH_4_ from H_2_ and CO_2_ could lead to the assembly of more complicated biomolecules. This hydrothermal scenario for the origin of life has been supported by many others, for example Wächtershäuser (1988) and Holm and Charlou (2001).

Hydrothermal vents are characterized by a number of specific physicochemical properties (Table 3a), which make them a favorable environment for prebiotic processes and possibly the emergence of life. High heat flux through the crust of early Earth (Kamber, 2015; Van Kranendonk *et al.,* 2015) suggests that hydrothermal activity would have been more widespread than at present. Fluid temperatures in modern hydrothermal vents are variable: up to >400°C for acidic fluid effluent from black smokers clustered atop oceanic spreading ridges (von Damm, 1990; von Damm *et al.,* 2003) to lower temperatures of 40–70°C for alkaline fluids issuing from more distal, off-ridge axis, white smokers (Kelley *et al.,* 2001). The greater heat flow in the Hadean crust may have produced temperature conditions too hot for the existence of white smoker-type vents (Sleep, 2000; Zahnle *et al.,* 2007).

**Table 3a.** Environments Available for the Emergence of Life

*System*	*Prebiotic chemistry*		*Emergence of life*	
*Properties*	*Advantages*	*Disadvantages*	*Advantages*	*Disadvantages*
**Submarine hydrothermal vents**				
Temperatures >100–150°C	Facilitates molecular interactions	Agitation can break up molecules		Temperatures too high unless the vents are inactive and flushed with lower-temperature seawater
Fluid dynamics	Low in porous edifice	High in vent throat	Diffusion of nutrients	Disruption if too high
pH—alkaline-acid	Favors prebiotic processes		Any pH for protocells	
Ionic strength	Variable		Salts necessary for protocells	
Energy sources	Heat; exothermal breakdown of organic molecules		Breakdown of organic molecules; redox reactions of reactive minerals; gradients (pH, temperature…)	
Mineralogy, *e.g.,* sulfides and various mafic/ultramafic minerals and their alteration products	Reactive surfaces favor molecule-organic interactions (Table 1)		Energy from redox reactions at mineral surfaces	
Element availability	CHNOPS		CHNOPS, Cu, Fe, Ni, Zn…	
Porous structures (minerals, edifices, *e.g.,* beehive structures)	Concentration of prebiotic components, compartmentalization		Protected environment for protocells	
Protection from external environment	UV protection, disruption caused by impacts		Protected environment—from UV, etc.	
Distribution of products	In hydrothermal fluid effluent		In hydrothermal fluid effluent	

The indigenous heat of hydrothermal systems and the heat produced by exothermic chemical reactions, including redox reactions occurring at the surfaces of reactive minerals within the hydrothermal vent edifices, are potential energy sources for prebiotic reactions occurring within the hydrothermal systems, as well as for primitive protocellular metabolisms. In prebiotic chemistry, we cannot define a temperature limit; nevertheless, there is competition between formation of a molecule and its destruction by hydrolysis. To displace equilibrium toward the formation of products, it is considered that the temperature would have to have been below 150°C. Moreover, at temperatures above 120°C the breakup of critical biomolecules, such as RNA, would have occurred (Kashefi and Lovley, 2003). We suggest, therefore, that temperatures must have been below 120°C to allow the accumulation of critical biomolecules.

In terms of fluid dynamics, within the vent throat itself, high Reynolds number flows (*i.e.,* turbulent flows) create flow instabilities, which would not encourage long-term mixing; however, within the porous hydrothermal structures themselves, turbulence would be much reduced. Hydrothermal fluids contain salts and organic molecules, essential for both prebiotic chemistry and for primitive metabolisms (Holm and Charlou, 2001; Pizzarello and Shock, 2010). The hydrothermal fluids interact with mafic and ultramafic rocks and their alteration products, as well as precipitate sulfide minerals, the reactive surfaces of which favor organic-mineral interactions that affect concentration, structural conformation, stabilization, and complexification of molecules (*e.g.,* Wächtershäuser, 1988; Russell and Hall, 1997). Hydrothermal edifices have the additional attribute that they are porous, their small cavities providing the possibility for the concentration of prebiotic components and eventual compartmentalization. Being submarine features, they provide protection from external stresses such as UV radiation and, to a certain extent, impacts that could destroy prebiotic molecules. Finally, submarine hydrothermal fluid effluent could have promoted the distribution of prebiotic products into seawater.

Russell and Arndt (2005) envisaged for the Hadean a situation similar to that of the present day, with active modern-style plate tectonics driving hot (∼400°C) hydrothermal vents on top of spreading ridges and cooler (∼40°C) vents occurring off-axis. However, given the different composition and rheology of the Hadean crust, plate tectonics in the modern fashion had probably not evolved in the Hadean era (*cf.* Breuer and Spohn, 1995; Kamber, 2015). Nevertheless, as a result of abundant volcanic activity driven by high heat flow (Sleep, 2000; Zahnle *et al.,* 2007), it is likely that hydrothermal cycling in the crust of the Hadean Earth was vigorous.

#### 1.3.2. Pumice rafts

Brasier *et al.* (2011, 2013) proposed rafts of pumice floating in the ocean as a location for the origin of life. Pumice has a number of properties that are favorable to prebiotic processes (Table 3b). Pumice can be described as a “frothy” type of lava formed during explosive volcanism that has a typically acidic composition with a high degree of silica polymerization (andesitic to rhyolitic) composition and, more rarely, a basic (basaltic) composition in which silica is less polymerized. During the Hadean, the crust had a primarily basaltic composition (Kamber, 2015); thus the volume of pumice produced during this period was likely small. We note, however, that some local felsic volcanism is recorded in Paleoarchean formations (*e.g.,* Lanier and Lowe, 1982; Lowe and Byerly, 1999; de Vries *et al.,* 2010), in which there is evidence for pumice (Westall *et al.,* 2006b; Brasier *et al.,* 2013). Although mostly composed of volcanic glass, pumice today contains small amounts of minerals, such as feldspar, pyroxene, amphibole, and zircon. Secondary minerals, for example zeolites, may be deposited in its cavities by percolating water. Pumice can also adsorb metals, organics, and phosphates, as well as host organic catalysts, such as zeolites and titanium oxides. As floating objects at the atmosphere-ocean interface, the temperature ranges experienced by pumice rafts are lower than those at subsea hydrothermal vents, with maximum temperatures probably not exceeding 70°C. During the Hadean, however, temperatures may even have been briefly freezing (*cf.* Russell and Arndt, 2005), and the gradients between the pumice and the surrounding Hadean seawater would have been very small, perhaps no more than several degrees. pH values in the pumice cavities can be variable, depending upon the original composition of the rock and the properties of the seawater permeating it. Ionic strength of the permeating fluids would be variable, depending upon the salt content of the seawater and the effects of subaerial desiccation (rafts of pumice can be “stranded” in the shallow-water tidal environment, permitting dehydration). Energy sources for prebiotic reactions could have been provided by heat, if the pumice rafts were in the vicinity of volcanic or hydrothermal activity, and possibly from ambient UV photolysis, although the mineral structure would act as protection against UV radiation. Pumice, *per se,* does not contain any native reduced carbon, although a small component of mantle origin might be envisaged. It can, however, naturally absorb organic carbon and phosphate (Brasier *et al.,* 2013). Titanium oxide and zeolite coatings within the vesicles are known catalysts in industrial processes (Brandes *et al.,* 1998; Brito *et al.,* 2004). Thus, where flushed perhaps by hydrothermal fluids in shallow-water settings, organic molecules could penetrate into the porous rock. The small, interconnected pores, on the order of microns to millimeters, could provide natural reaction flasks for the complexification of prebiotic molecules, which could continue over the long life span of the pumice clast (Brasier *et al.,* 2011). The rock would protect products of processes occurring in the pores from UV radiation, and the very low density of pumice could help protect it during impact events by simply floating away from disturbance under the shock force or even ejection into the atmosphere to be redeposited elsewhere. Indeed, save for its volumetrically limited, and probably localized, generation, pumice provides an excellent means of distributing prebiotic products throughout the ocean.

**Table 3b.** Environments Available for the Emergence of Life

*System*	*Prebiotic chemistry*		*Emergence of life*	
*Properties*	*Advantages*	*Disadvantages*	*Advantages*	*Disadvantages*
**Pumice**				
Temperatures <70°C	Temperatures conducive to prebiotic chemistry; occasional freezing possible		Temperatures above freezing conducive to protocell activity	
Fluid dynamics	Low in porous edifice		Diffusion of nutrients	
pH—variable, depending on the composition of the pumice and the properties of the seawater		Does not favor ribose formation	Any pH for protocells	
Ionic strength	Variable, depending upon ambient seawater		Salts necessary for protocells	
Energy sources	Heat if in vicinity of volcanic or hydrothermal edifices		Energy from redox reactions at surfaces of volcanic glass; gradients (*e.g.,* pH, *T*)	
Mineralogy	Volcanic glass of felsic to more rarely basaltic origin		Energy from redox reactions at surfaces of volcanic glass	
Element availability		No CHNOPS but could adsorb organics from seawater	Na, K, some transition metals, Fe	No CHNOPS but could adsorb organics from seawater
Porous structures	Micro-pores could act as mini reactors; concentration of prebiotic components, compartmentalization		Protected environment for protocells	
Protection from external environment	Protection from UV and disruption caused by impacts		Protected environment—from UV, etc.	
Distribution of products	Floating on ocean surface		Floating on ocean surface	

#### 1.3.3. Subaerial geysers

Although most hypotheses for the origins of life have considered marine geological settings, an ever-increasing number of propositions for the origin of life in a terrestrial system are being considered (*i.e.,* in an atmosphere that was anoxic). The characteristics of hot spring geysers are summarized in Table 3c. Geysers are ephemeral subaerial hydrothermal vents around which fluids deposit minerals and mineraloids dictated by the composition of the underlying formations, through which the heated subsurface water rises. For example, siliceous and carbonate deposits form at Yellowstone National Park, depending on the source of the fluids. On a largely basaltic Hadean Earth, the deposits from geysers would have been mainly siliceous in composition rather than carbonate, similar to siliceous sinters in Yellowstone, Iceland, Kamchatka, or New Zealand (Campbell *et al.,* 2015, and references therein). Physical variations in vent-area hydrodynamics within subaqueous and subaerial environments produce stratiform, spicular, beaded, nodular, and columnar geyserite varieties (*e.g.,* Walter *et al.,* 1996; Braunstein and Lowe, 2001; Jones and Renaut, 2003; Hinman and Walter, 2005; Campbell *et al.,* 2015). However, both ancient and modern sinter deposit textures are invariably strongly influenced by biology and thus constitute a compromised analogue to their Hadean precursors (*e.g.,* Konhauser and Ferris, 1996; Jones *et al.,* 2001; Jones and Renaut, 2003). Geyserites are rocks formed around high-temperature hot springs, the exiting water and steam exceeding the boiling point but cooling to ambient temperatures with distance from the vent (even to freezing in apposite environments). Water exiting the vents would be exposed to solar radiation, leading to hydrogen peroxide production (Wilson *et al.,* 2000). On the anoxic Hadean Earth, peroxide would have oxidized organic molecules and thus potentially hindered prebiotic chemistry and cellular life, if the latter existed. Fluids in the vents themselves would have mixed vigorously and turbidly, but the intensity of mixing would have decreased away from the vent in the surrounding mineral deposits. pH values of the fluids are variable, ranging from alkaline to acidic (Cady and Farmer, 1996; Walter *et al.,* 1996; Braunstein and Lowe, 2001; Hinman and Walter, 2005; Campbell *et al.,* 2015); generally, the ionic concentration of the hydrothermal fluids depends on their interaction with the formations through which they pass (Rowe *et al.,* 1973; Livo *et al.,* 2007). Hydrothermal alteration of the underlying rock formation will produce a suite of alteration minerals. For example, at Yellowstone, the alteration mineral suite includes quartz, adularia, illite, sulfide, alunite, and kaolinite (*e.g.,* Livo *et al.,* 2007). By contrast, ∼3.5 Ga springs from the Pilbara precipitated silica and barite (Djokic *et al.,* 2017), perhaps approximating the Borate Springs of the Puga Valley, India.

**Table 3c.** Environments Available for the Emergence of Life

*System*	*Prebiotic chemistry*		*Emergence of life*	
*Properties*	*Advantages*	*Disadvantages*	*Advantages*	*Disadvantages*
**Subaerial geysers**				
Temperatures < ∼130°C; possibly freezing	Facilitates molecular interactions; freezing temperatures stabilize ribose and promote catalytic activity of RNA		Cellular activity up to 120°C	
O_2_ production from production of H_2_O_2_		Will destroy organic molecules		Will destroy organic molecules
Fluid dynamics	Low in porous edifice surrounding geyser	High in vent throat	Diffusion of nutrients	Disruption of structures in vent throat
pH—alkaline-acid	Favors prebiotic processes	Acidic pH unfavorable for ribose	Any pH for protocells	
Ionic strength	Variable		Salts necessary for protocells	
Energy sources	Heat; exothermal breakdown of organic molecules		Breakdown of organic molecules; redox reactions of reactive minerals; gradients (pH, temperature…)	
Silica (carbonates today depending upon underlying lithology)	Silica surfaces favor molecule-organic interactions (Table 1)		Energy from redox reactions at country rock mineral surfaces	
Element availability	CHNOPS from hydrothermal fluids		CHNOPS, Cu, Fe, Ni, Zn…	
Porous structures (minerals, edifices, *e.g.,* beehive structures)	Concentration of prebiotic components, compartmentalization		Protected environment for protocells	
Protection from external environment	Some UV protection in porous sinter deposits	Desiccation and exposure to UV	Some protection from UV, etc.	Desiccation and exposure to UV
Distribution of products		Difficult		Difficult

Potential energy sources for prebiotic reactions stem from heat, exothermal organic breakdown, and possibly UV radiation, unless the location was sufficiently well protected by the mineral precipitate, while oxidation of organic matter, redox reactions (minerals from the country rock), and gradients in pH and temperature could fuel primitive metabolisms. The elements essential for prebiotic chemistry and early life could have been made available through hydrothermal circulation in the crust (from either Fischer-Tropsch-type synthesis or from fluid inclusions in ultramafic rocks; *cf.* Shock *et al.,* 2002; McDermott *et al.,* 2015) and eventually from recycled meteoritic carbon. Sulfur and transition metals could also be available for primitive metabolisms, depending upon the source rocks.

A number of reasons have been put forward for suggesting that subaerial environments are conducive to the emergence of life. Mulkidjanian and colleagues noted that the K^+^/Na^+^ ratios of terrestrial freshwater fields are synonymous with those of modern cellular cytoplasm (Mulkidjanian *et al.,* 2012a, 2012b; Dibrova *et al.,* 2015). Deamer and Georgiou (2015) highlighted wetting-drying cycles, a characteristic repetitive process of modern terrestrial hot springs, as greatly enhancing the ability of these systems to concentrate and polymerize mononucleotides in the residual deposits of springs. These polymerized nucleotides could stabilize protocell membranes, control pore insertion that provides access to nutrients, and catalyze metabolisms (Damer and Deamer, 2015). Broadly, proponents of these systems note their lack of divalent cations relative to the oceans and suggest that their relatively small, well-bounded sizes increase the chance of concentrating prebiotic molecules; that is, they are, at times, more concentrated than oceans. Geyser-produced rocks have multiple surface heterogeneities (Walter *et al.,* 1996; Jones *et al.,* 2001, 2005; Campbell *et al.,* 2015) that could provide mineral loci for the concentration of molecules during the drying stage of each wetting-drying cycle (Damer and Deamer, 2017). Moreover, the high presence of volatiles, for example ammonia, phosphorus, borate, and cyanide, facilitates polymerization of nucleotides by condensation reactions under the influence of solar energy (Mulkidjanian *et al.,* 2003, 2012a; Dibrova *et al.,* 2015).

Subaerial environments have attracted further attention for their possible sources of elements, such as boron and molybdenum, shown in the laboratory to help the formation of ribose, one of the key components of RNA (*e.g.,* Benner *et al.,* 2008). Benner's group (Neveu *et al.,* 2013) thus consider boron-containing minerals and borate exposed at the Earth's surface, a situation considered by Grew *et al.* (2011) to be tectonically unlikely.

Another hypothesis by Ebisuzaki and Maruyama (2017) proposes a nuclear geyser model for the geological setting of the origin of life. This involves a geyser driven by a natural nuclear reactor, claimed to have been common on the Hadean Earth, due to the abundance of ^235^U as nuclear fuel. The nuclear geyser supplies the following: (1) high-density ionizing radiation to promote chemical chain reactions; (2) a system to maintain the circulation of material and energy, which includes cyclical environmental conditions that enable the production of complex organic compounds; (3) a temperature lower than 100°C so as not to break down macromolecular organic compounds; (4) a locally reducing environment, which depends on the lithology exposed along the geyser wall; and (5) a container to confine and accumulate volatile chemicals. One major problem with this hypothesis is that such nuclear reactors, as are known from the geological record, could only have appeared after the oxidation of Earth about 2.4 Ga, since uranium can only be mobilized and concentrated in an oxidizing environment.

Although the porous structure of geyserite deposits could serve as protection from UV radiation, the flux of UV to early Earth was much higher than today (Cockell and Raven, 2004). These subaerial environments are also highly ephemeral. Consequently, the time permitted for the evolution, flourishing, and migration to the oceans of prebiotic molecules or cellular life (see Damer and Deamer, 2017) would likely have been very short. This temporal aspect is compounded by the fact that the surface environments that did exist would have been subjected to impacts from continued planetary accretion. Although there is debate about the reality of the hypothesized Late Heavy Bombardment (Ryder *et al.,* 2000; Koeberl, 2006), which appears to have been more benign than previously supposed (Sleep, 2016; Zellner, 2017), it is clear that these high-energy impacts would have resulted in destruction of subaerial environments, even taking into account the fact that, since the Hadean-Archean Earth was largely an oceanic planet, subaerial environments would not have been very common, although it is likely they were continually renewed. Indeed, there is evidence only for rare emergent landmasses even at ∼3.5 Ga (Van Kranendonk and Pirajno, 2004; Van Kranendonk, 2006; Westall *et al.,* 2006b; Djokic *et al.,* 2017); when extrapolated back to the early Hadean, such landmasses may not have been emergent, but rather evolving submarine “protocontinents” (*cf.* Kamber, 2015).

#### 1.3.4. Volcanic-hosted splash pools

Coastal volcanic environments have also been invoked as potential locations for the formation of biomolecules, such as photopigments (Fox and Strasdeit, 2013). Arguing that these kinds of environments would have been plausible for early Earth, Fox and Strasdeit (2013) imagined a stepwise scenario involving two different, but contiguous, environments: the condensation of amino acid–containing salts on hot lava to produce pyrrols, followed by the transport of pyrrols in vaporized seawater to cooler volcanic-hosted rock pools where they condensed and became oligomerized. Hydrochloric acid that formed in the acidic vapor was an important contributor, as were Fe^2+^ and Mg^2+^ ions liberated from acid-leached basalts that contributed to the formation of metal-oligopyrrols. Noting the high UV conditions (especially shorter UV wavelengths) prevalent on early Earth, Fox and Strasdeit (2013) underlined the importance of pigments in the protection of early cellular life. However, recent experiments have shown that prebiotic molecules, such as amino acids, have a low rate of survival in basaltic rocks containing ferrous iron when exposed to UV (dos Santos *et al.,* 2016).

We have summarized the physicochemical characteristics of volcanic-hosted splash pool environments in Table 3d. Temperatures can range widely; they are high on the freshly erupted lavas and low in the rock pools, being even freezing if early Earth was frozen periodically. Fluid dynamics would be variable, low in the salt crusts on the hot lava and periodically high in the tidal splash pools, which could lead to disruption of molecular bonds. The pH would be low, necessary for oligopyrrol synthesis but not favorable for ribose formation. Ionic strengths in the salt crusts would be high. Energy would come from heat from the lava and UV flux to the surface of Earth, both potentially deleterious for life. Redox reactions at the surfaces of mafic/ultramafic minerals in the crust would have driven primitive metabolism. The mineralogy would be derived from the basaltic rocks, that is, pyroxene, olivine, feldspars, accessory oxides, and their alteration products (phyllosilicates, sulfides, etc.). The essential elements would have come from mantle organic compounds and organic compounds in the seawater, with S, Fe^2+^, Mg^2+^, and other transition elements coming from the altered basalts. The salt crust would have provided a porous structure for compartmentalization of prebiotic minerals, as would the altered volcanic rocks in the rock pools. While the salt crusts could provide protection from UV (Cockell *et al.,* 2010), protocells in the rock pools would probably be negatively affected by the UV flux on the Hadean Earth. The products formed in the pools would be distributed by tidal flow and splashing. These coastal environments, like the subaerial geysers, would have suffered from instability and impacts. Like all the aforementioned environments, they could have hosted certain prebiotic processes, but it is less likely that life appeared and flourished in such very short-lived rock pools.

**Table 3d.** Environments Available for the Emergence of Life

*System*	*Prebiotic chemistry*		*Emergence of life*	
*Properties*	*Advantages*	*Disadvantages*	*Advantages*	*Disadvantages*
**Volcanic coastal environments (seawater heated by hot lava and rock pools)**				
Temperatures, high on lavas (200°C), low in rock pools (<100°C), to possibly freezing depending on early Earth scenario	Relevant for prebiotic chemistry		Temperatures <120°C permissive of cellular activity	
Fluid dynamics—variable	Variable due to tidal energy, splashing but negligible salt crusts on hot lava	Too much agitation in tidal pools could break up the molecules	Permissive of cellular activity	
pH acid	Necessary for oligopyrrol synthesis	Does not favor ribose formation and stability	Any pH for protocells	
Ionic strength	High		Salts necessary for protocells	
Energy sources (heat, UV, redox)	Heat necessary for salt precipitation and pyrrol volatilization; UV fluxes to lava surface and rock pools would have been high	Heat and UV not good for stability of ribose	Redox reactions on reactive minerals; gradients (pH, temperature…)	UV deleterious to cellular life
Mineralogy, *e.g.,* mostly basalt lava (pyroxene, olivine, feldspars, accessory oxides) and their alteration products	Redox reactions important for oligopyrrol formation		Energy from redox reactions at mineral surfaces	
Element availability	CHNOPS from organics in water and mantle carbon in basalts; Fe^2+^ and Mg^2+^ from altered basalts		S and transition elements Cu, Fe, Ni, Zn…	
Porous structures (minerals, edifices, *e.g.,* beehive structures)	Altered basalts in rock pools, mineral porosity, sediments		Protected environment in rock pools for protocells	
Protection from external environment		No protection from UV, disruption caused by impacts or volcanic eruptions		No protection from UV, disruption caused by impacts or volcanic eruptions
Distribution of products	Possible if pool is tidal		Possible if pool is tidal	

### 1.4. Summary of the geological context for the origin of life

Above, we have summarized the very limited information available concerning the geological environments of the Hadean Earth (*cf.* Russell and Arndt, 2005; Kamber, 2015; Van Kranendonk *et al.,* 2015). The geological boundary between the Hadean and the Eoarchean was thought to be sharp and strongly influenced by the hypothesized Late Heavy Bombardment as well as tectonic overturn (Kamber, 2015). However, the recent suggestion of a period of prolonged bombardment between 4.2 and 3.4 Ga rather than a short, sharp spike between 4.0 and 3.85 Ga (Zellner, 2017) might suggest a more gradual crustal evolution. We briefly described the different scenarios proposed for the emergence of life, ranging from subaerial springs (*e.g.,* Mulkidjanian *et al.,* 2012a, b), volcanic island coastal splash pools (Fox and Strasdeit, 2013), pumice rafts (Brasier *et al.,* 2011, 2013), to submarine hydrothermal vents (Baross and Hoffman, 1985; Russell and Hall, 1997; Martin *et al.,* 2008; Sleep *et al.,* 2011; see also review in Dass *et al.,* 2016) and addressed their significance with respect to what could have been the Hadean environment.

**FIG. 1.**
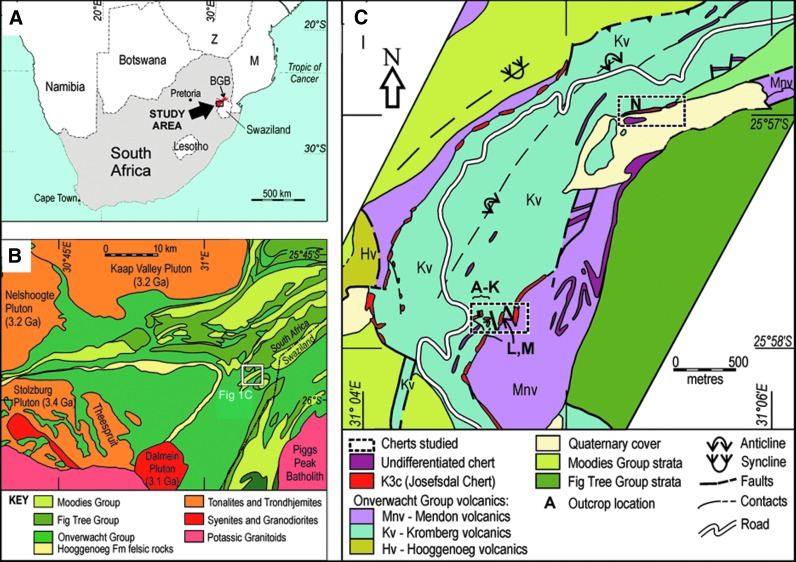
(**A**) Location of the study area within South Africa. (**B**) General geological map of the Barberton Greenstone Belt showing location of the JC (white box). (**C**) Detailed geological map of the thin JC sediment exposures (red) within large thicknesses of mafic and ultramafic volcanics. Adapted from Westall *et al.* (2015a).

In the following, we describe another environment in which prebiotic chemistry could have occurred and life may have emerged: the layer of porous, volcanic, reactive sediments at the interface between mafic-ultramafic Paleoarchean crust and seawater. These processes involved the interaction of ubiquitous hydrothermal fluids with volcanic sediments charged with carbonaceous matter of multiple origins at scales ranging from outcrop- (point source venting) to microscopic-scale infiltration of the porous sediments. This is a novel and hitherto unrecognized phenomenon that we document in Early Archean 3.33 Ga sediments from the Josefsdal Chert (JC), Barberton Greenstone Belt, South Africa (Fig. 1). Our observations describe field- to microscopic-scale phenomena, and we suggest that similar hydrothermal-sedimentary interface phenomena could have been common on the ocean-dominated Hadean Earth, despite possible differences in the global-scale geological situation between the Hadean and the Paleoarchean. Our observations thus add a new and significant spatial dimension to the geological setting of the complexification of the organic building blocks of life, a key progression in the evolutionary continuum leading to the origin of life itself.

## 2. Early Earth Sediments and Hydrothermal Perfusion^1^

### 2.1. Sedimentology

**FIG. 2.**
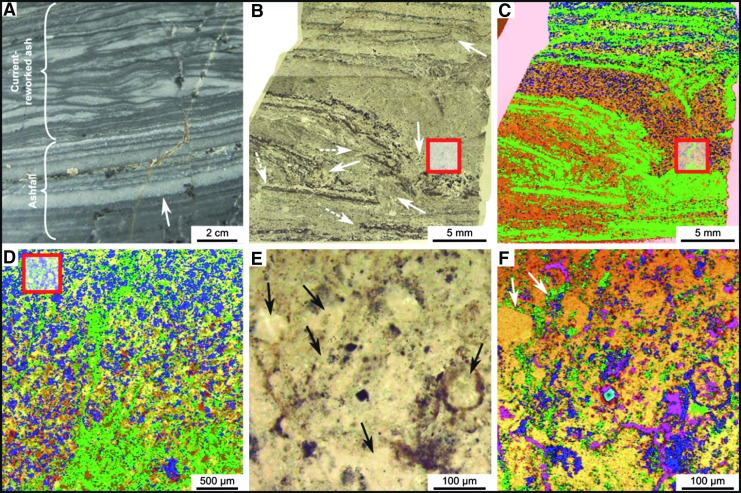
Early Archean, laminated shallow marine volcanogenic sediments interacting with pervasive hydrothermal effluent from the 3.33 Ga JC. (**A**) Field photograph of alternating ash (light) and carbonaceous (dark) layers. (**B**) Photomicrograph of a thin section from the same facies documenting layers disrupted, through soft sediment deformation, by infiltrating hydrothermal fluids (arrowed). Red box outlines detail in (D). (**C**) Raman map of (B), showing carbon (green) within the silicified matrix (orange, quartz); anatase (blue) represents altered volcanic clasts. Red box outlines details in (D). (**D**) Raman map (red boxes in B, C) showing carbon (green) intermixed with volcanic particles (represented by alteration phases: anatase, blue; muscovite, pink); the quartz matrix (yellow/orange) represents the silica precipitated by hydrothermal fluids. Optical image (**E**) and Raman map (**F**) are details of the red box in (D) showing carbon (green) coating volcanic particles (arrowed), which have been replaced by muscovite (pink), anatase (blue), and quartz (yellow/orange). Additional minerals: magnetite, light blue; rutile, red.

The Josefsdal Chert (JC) consists of several layers of volcanic sediments deposited in a shallow-water environment on top of basalt lava and covered again by basalt lava (Westall *et al.,* 2015a). The sedimentary succession is divided into four facies representing sedimentation in shifting upper offshore to foreshore (beach) environments. Depending upon the environment of deposition, the sediments exhibit varying textures, grain size, and porosity (*e.g.,* Fig. 2). Sediments deposited under a dynamic regime, for example storm conditions, are sand-sized (63–500 μm), well-sorted, and porous. Sediments deposited in quiescent regimes are finer-grained, silt to fine sand–sized (<100 μm), more poorly sorted, and less porous. Ashfall into quiet water environments produced graded layers of sand-sized grains (>250 μm) to silt and fine detrital carbonaceous matter (<63 μm) that were intermittently reworked into rippled structures by wind and tidal or current reworking (Fig. 2A).

The volcanic particles were altered to phyllosilicate (*e.g.,* smectite) and anatase before silicification of the sediments (silicification explained below). This is demonstrated in Figs. 2E–2F in which individual volcanic particles in the transmitted light micrograph (Fig. 2E) are shown to be composed of muscovite (pink color, the muscovite being the metamorphosed form of original smectite) or are outlined by anatase crystallites (blue) in the Raman mineralogical map in Fig. 2F. All were subsequently infiltrated and replaced to varying degrees by silica.

**FIG. 3.**
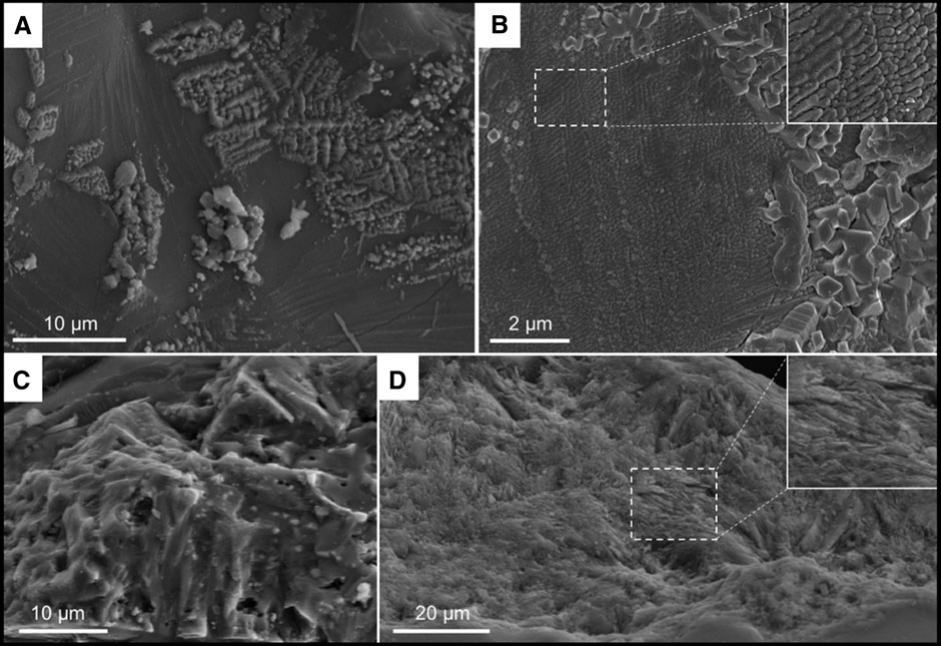
Crushed volcanic rocks (mixture of East Pacific Rise basalt and komatiite) before and after corrosion in artificial seawater (starting pH = 6.24). (**A**) Scanning electron microscope view of the surface of basalt glass with skeletal pyroxene crystals showing mineralogical and morphological variability on the micron scale. (**B**) Detail of the volcanic glass surface (with inset) documenting submicron-scale morphological heterogeneity. (**C**) Sample from the same crushed volcanic rocks after 15 days of corrosion (ending pH = 7.1). The pyroxene crystals show pits and pores ranging in size from submicron to a few microns. (**D**) Phyllosilicate-coated surface of a volcanic glass shard corroded for 15 days (ending pH = 7.1).

**FIG. 4.**
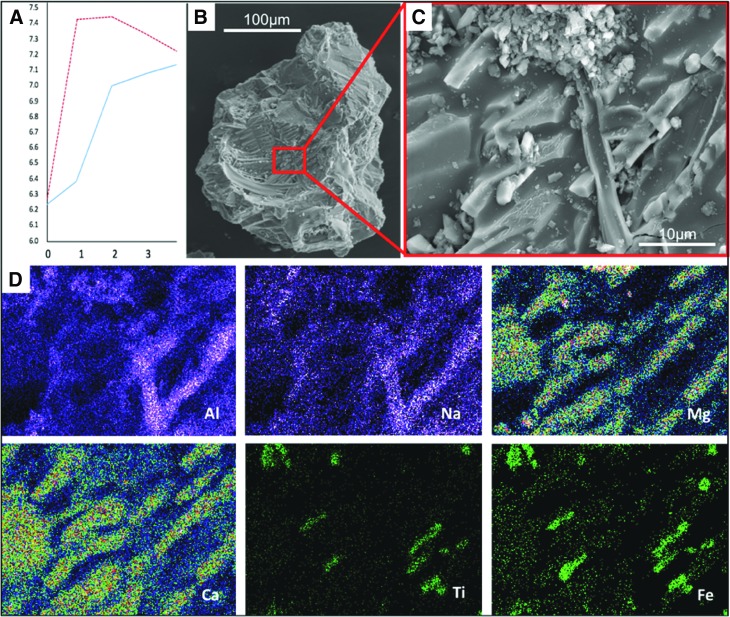
Corroded volcanic grains from a mixture of East Pacific Rise basalt and komatiite (as in Fig. 3). (**A**) Changing pH during the first 3 days of corrosion of a mixture of East Pacific Rise basalt and komatiite in artificial seawater with a starting pH of 6.24. The red curve denotes pH changes in the sediment, blue curve changes in the overlying seawater. The rapid change in pH to more alkaline conditions from an initial weak acid is ascribed to the reaction of this seawater with particularly reactive minerals in the volcanic grains; the pH gradient in the pore spaces of volcanic sediment can thus be initiated in days. (**B**, **C**) Scanning electron micrographs of an ex-gas bubble in a pyroxene grain, documenting variable surface textures including porosity and protrusions associated with compositional variability (D). (**D**) EDX elemental maps showing two main phases: needle-shaped crystals of pyroxene (Na, Mg, Al, Ca, trace Fe; Si not shown) co-precipitated with ilmenite (FeTiO_3_).

To observe incipient alteration phenomena on basalt particles, we exposed a mixture of crushed basalt and komatiite for 4 days at 50°C to analog Hadean seawater having an initial pH of 6.24 (Figs. 3–4). Even before alteration, the crushed volcanic particles exhibited micron- to submicron-scale morphological heterogeneity, as documented in Figs. 3A–3B. During alteration in the slightly acidic seawater, there was an initial, rapid rise in pH from 6.24 to 7.4 within the sediments (red curve in Fig. 4A), which decreased gradually to equilibrium around 7.1. Alteration phenomena in the volcanic particles include the formation of pores 1–3 microns (Fig. 3C) and phyllosilicates (Fig. 3D). Figures 4B–4C, which show a former gas bubble in an altered pyroxene grain, document micron-scale cavities, depressions, and irregularities at its surface. These morphological heterogeneities are accompanied by similarly scaled elemental variations related to the diverse minerals exposed and altered on the particle surface. EDX elemental maps show two micron-scale phases; needle-shaped crystals of pyroxene (Na, Mg, Al, Si, Ca, trace Fe) co-occurred with ilmenite (FeTiO_3_).

### 2.2. Evidence for hydrothermal activity

Physical evidence for hydrothermal activity and infiltration of silica-rich hydrothermal fluids consisted of visible macroscopic- to microscopic-scale hydrothermal veins and confirmation of contemporaneous, lateral, and vertical infiltration of fluids under pressure into the sediments. Mineralogical evidence included pervasive silicification of the entire sedimentary deposit and secondary mineralization (alteration minerals, hydrothermally precipitated minerals, such as siderite). Geochemical evidence was revealed by bulk analyses and *in situ* elemental distribution.

#### 2.2.1. Physical evidence

**FIG. 5.**
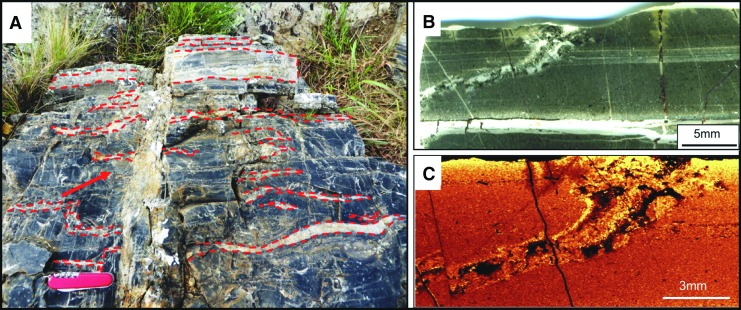
Macroscopic to microscopic hydrothermal veins. (**A**) Late diagenetic, vertical hydrothermal vent (full arrow) cross-cutting hummocky-swaley storm deposits in the JC (Facies A of Westall *et al.,* 2015a). Note also infiltrations of hydrothermal chert, emanating from the central vent, parallel to the sediment layering (outlined by dotted red lines). (**B**) At the thin-section scale, this optical micrograph shows a hydrothermal veinlet cross-cutting finely laminated, fine-grained sediments. (**C**) Raman map of carbon distribution in the sediment and in the vein shown in B); brighter color indicates higher concentration, *i.e.,* carbon is at its highest concentration when entrained within the vein.

Contemporaneous and post-lithification hydrothermal conduits and vents visible at the outcrop and the microscopic scale (Fig. 5) indicated point sources of fluid entry. Figure 5A shows a large, vertical, hydrothermal intrusion cross-cutting layers (almost invisible) of highly silicified hydrothermal sediments. Chert layers parallel to the bedding plane (dashed red outlined layers) represent the intrusion of pressurized hydrothermal fluids into partially lithified and still slightly deformable layers of volcanic sediments, provoking a swale-like undulating structure (as in the lowermost intrusion of Fig. 5A).

On the microscopic scale, Figs. 5B–5C document contemporaneous, forceful intrusion of hydrothermal fluids into cohesive but not lithified, fine-grained sediments. The borders of the intrusion are not straight, as would be the case in brittle, post-lithification intrusion (*e.g.,* the large vertical vein crosscutting the sediments in Fig. 5A), but rather are irregular and slightly blended with the surrounding sediments. The miniature intrusive feature broadens toward a bedding plane surface and terminates at the surface in a funnel-shaped exit structure (microscopic vent). The hydrothermal fluids in this vein transported reduced carbon (see Raman map in Fig. 5C), which was both deposited on the walls of the vein and expulsed with hydrothermal fluids into the seawater where it was deposited as floccular structures within the sediment (not shown).

Figures 2B–2D document mixing of internal layers of the volcanic sediments by fluids under pressure. The sediment layers, outlined in black, were disrupted and mixed (Fig. 2B, white arrows) as highlighted in the Raman mineralogical maps (Figs. 2C–2D). On a scale of millimeters to microns, carbonaceous layers (colored green in Fig. 2) are thoroughly mixed with the groundmass representing altered (pseudomorphed) volcanic grains (blue for anatase and yellow for quartz) in a quartz matrix (yellow to orange).

#### 2.2.2. Mineralogical evidence

**FIG. 6.**
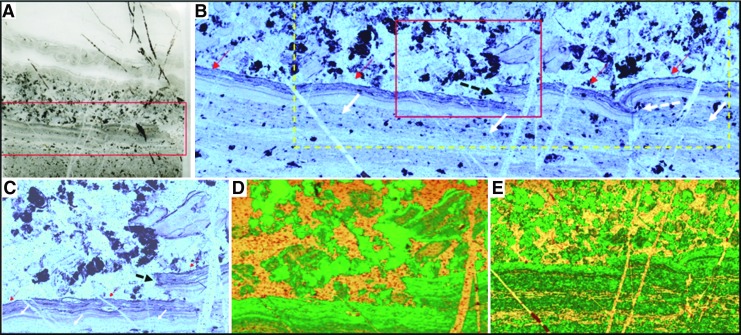
Silica gel-like sediment from the 3.33 Ga JC. (**A**) Scanned thin section slide of a deposit of hydrothermal silica containing carbonaceous clots and layering. (**B**) Detail of red box in (A) showing fine-scale carbonaceous layering in the lower part of the image and a mottled carbonaceous texture in the upper part. The top of the layered section shows plastic deformation (dashed white arrow) and tearing, indicative of disruption by the dynamic flow of hydrothermal fluids. Red arrows indicate the cohesive layer above the plastically deformed layer, and solid white arrows indicate detrital sedimentation below. The red box denotes the detail in (C) and the Raman scan in (D), while the yellow box denotes the area of the Raman map in (E). (**C**) Detail showing tearing of the cohesive surface of the finely laminated layer (black arrow). (**D**, **E**) Raman maps demonstrate that the sample consists of only quartz (yellow-orange) and carbon (green).

The top of the hydrothermally intruded outcrop in Fig. 5A is characterized by parallel layers of light-colored, nearly translucent silica. While some of the layers represent penecontemporaneous lateral intrusions of hydrothermal fluids into semi-lithified sediment, other layers display microscopic textures that suggest deposition as a chemical sediment, that is, as a silica gel (Fig. 6). Compositionally, the translucent chert consists of silica and carbon only, with no evidence for detrital volcanic input (see Raman maps in Figs. 6C–6D). This sediment consists of several layers exhibiting different textural and depositional characteristics. Highlighted in the red box in the central part of the scanned thin section in Fig. 6A, and enlarged in Fig. 6B (with accompanying Raman map, Fig. 6E), is a portion of the sediment in which a finely laminated layer lies beneath a mottled layer. Very fine layers of carbon outline the lower part of the sedimentary layering; the conformity of the layer indicates passive settling. These sediments are matrix-supported, that is, supported by silica. The top of the fine sedimented layers, on the other hand, consists of a packet of thin wrinkly layers of carbon (red arrows in Figs. 6B–6C) that are subparallel to the sediment bedding planes and show cohesive behavior and plastic deformation in the form of tear and roll-over structures (dotted black arrows in Figs. 6B–6C). Following the interpretations of Westall *et al.* (2015a), the packet of wrinkly layers represents a silicified phototrophic biofilm (*nota bene,* for the purposes of this paper, the origin of the carbonaceous matter—biogenic or abiogenic—in the Josefsdal sediments is irrelevant; of importance here is the documentation of the interaction between hydrothermal fluids and carbonaceous sediments).

Above the torn biofilm is a chaotic layer of mixed carbonaceous clots and torn fragments of the underlying phototrophic biofilm, again in a matrix-supported siliceous sediment. The association of the chaotic layer with evidence of physical deformation of the phototrophic biofilm suggests turbulent flow. Adjacent to the sampling location is a growth fault (not shown) which appears to have been the conduit for the hydrothermal fluids that deposited as a silica gel-like sediment on top of the immediately surrounding sedimentary surfaces at the same time as laterally penetrating, partially lithified sediments in bedding-parallel, sill-like intrusions. Precipitation of silica as a gel from supersaturated hydrothermal fluids that form a chemical sediment has previously been suggested for rocks of a similar age and appearance from the Onverwacht Group by Ledevin *et al.* (2014).

Silicification, which occurred on timescales ranging from contemporaneous to early diagenetic to late diagenetic, provides further evidence for hydrothermal influence on the JC sediments. Penecontemporaneous silicification is documented by soft-sediment deformation due to intruding silica-rich fluids, as shown in Fig. 2B, and documented in Westall *et al.* (2015a) by fossilization of a living, *in situ* phototrophic biofilm by silica (Westall *et al.,* 2006a, 2011). Partial to total replacement of the altered volcanic particles by silica (Figs. 2E–2F, black arrows; Fig. 6) took place rapidly as indicated by the lack of compaction of any of the particles, especially the plastically deformable ones, such as carbonaceous clots. Silicification of the sediments would have occurred through the precipitation of silica gel in the pore spaces from silica-enriched fluids (*i.e.,* seawater mixed with hydrothermal silica and pore waters infiltrated by silica-rich hydrothermal fluids).

The lower layers of the JC, stratigraphically just above the underlying basalt, are more highly silicified (up to 99% SiO_2_) than the upper layers (up to 96% SiO_2_), with the variable degree of silicification being observable at outcrop scale. The lower JC layers appear black, and their sedimentary structures are almost completely obfuscated owing to the very significant degree of silicification. In these layers, the rocks are also suffused by myriad small to large, late-stage hydrothermal silica veins. Toward the top of the outcrop, although the silica contents are still high (up to 96%), the sedimentary structures are perfectly preserved and readily visible, while late-stage hydrothermal veins are rarer (Fig. 5A).

**FIG. 7.**
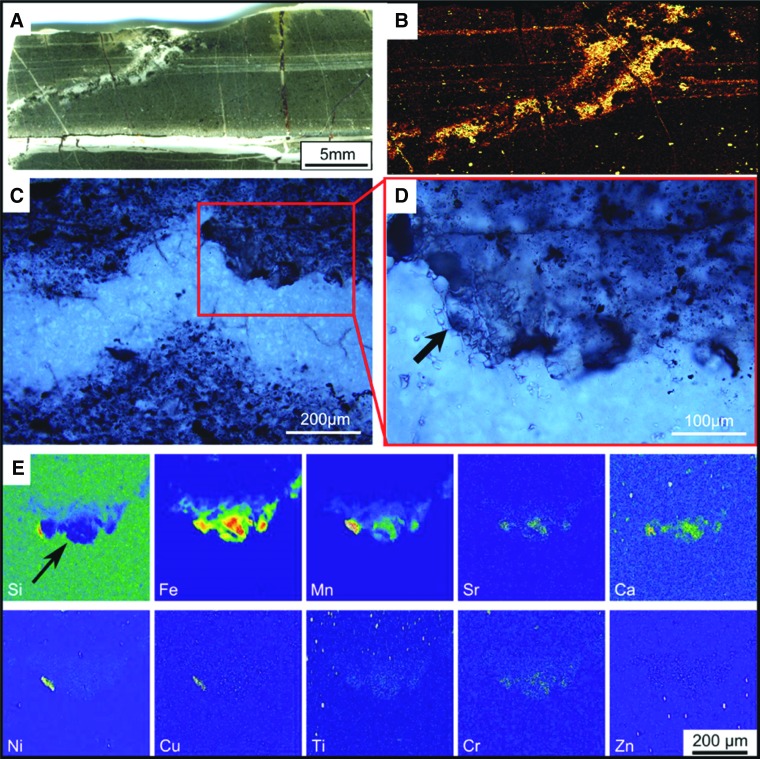
Hydrothermal veinlet in fine-grained carbonaceous sediments from Josefsdal. (**A**) Optical micrograph showing a hydrothermal veinlet cross-cutting finely laminated, fine-grained sediments. (**B**) Raman map of the siderite distribution in the sediment and in the veinlet; brighter color indicates higher concentration. (**C**, **D**) Optical micrograph views of a siderite (FeCO_3_) and rhodochrosite (MnCO_3_) co-precipitate at the edge of the veinlet shown in (A). Red box in (C) shows location of detail in (D). Arrow in (D) points to the same location as the arrow in (E). (**E**) PIXE elemental maps (beam size: 2 μm; map size: 500 × 500 μm; resolution: 256 × 256 pixels; 11 h acquisition time) of area denoted by red box in (C) document concentrations of other elements associated with the siderite/rhodochrosite precipitation, including Mn, Sr, Ca, Ni, Cu, Ti, Cr, and Zn scavenged from the hydrothermal fluids.

Siderite is co-precipitated with the silica in the finer-grained, more poorly sorted sediments close to hydrothermal vents and in contemporaneous hydrothermal veins themselves. Co-precipitation with silica is indicated by the fact that siderite is the only mineral phase not to be silicified (Figs. 7C–7E). The precipitation of siderite crystals stands testament to an environment infused with CO_2_, having a high inorganic carbon content, and variable pH, as modeled in Table 4.

**Table 4.** Results of the PHREECQ-Modeled Runs for Mineral Precipitation in Hadean Seawater

*PCO_2_*	*T (C)*	*Final pH*	*[Ca]_T_ (m/L)*	*[Fe(II)]_T_ (m/L)*	*SI (calcite)*	*SI (siderite)*	*Model*
1.0	50	7.99	5.02E-04	1.10E-07	1.01	−1.29	1
3.2	50	7.70	6.55E-04	3.29E-07	1.1	−1.04	1
10.0	50	7.44	9.48E-04	9.55E-07	1.19	−0.83	1
100.0	50	7.04	2.14E-03	6.10E-06	1.42	−0.43	1
1.0	100	7.08	5.18E-05	5.93E-07	−0.41	−0.59	1
3.2	100	6.81	5.35E-05	1.68E-06	−0.41	−0.37	1
10.0	100	6.56	5.44E-05	4.60E-06	−0.47	−0.17	1
100.0	100	6.14	5.37E-05	2.99E-05	−0.68	0.18	1
1.0	50	7.28	2.85E-06	1.87E-06	−1.37	−0.7	2
3.2	50	7.12	3.86E-06	3.98E-06	−1.3	−0.54	2
10.0	50	7.00	5.88E-06	7.73E-06	−1.14	−0.35	2
100.0	50	6.87	9.25E-06	1.78E-05	−0.84	0.05	2
1.0	100	6.74	5.38E-05	2.24E-06	−0.43	−0.31	2
3.2	100	6.54	5.44E-06	4.98E-06	−0.48	−0.16	2
10	100	6.36	5.45E-05	1.11E-05	−0.55	−0.01	2
100.0	100	6.04	5.39E-05	5.10E-05	−0.75	0.29	2

T = total concentration.

Model 1 = Initial equilibration with atmospheric CO_2_ followed by equilibration with minerals.

Model 2 = Equilibration with CO_2_ and minerals simultaneously.

**Figure:**
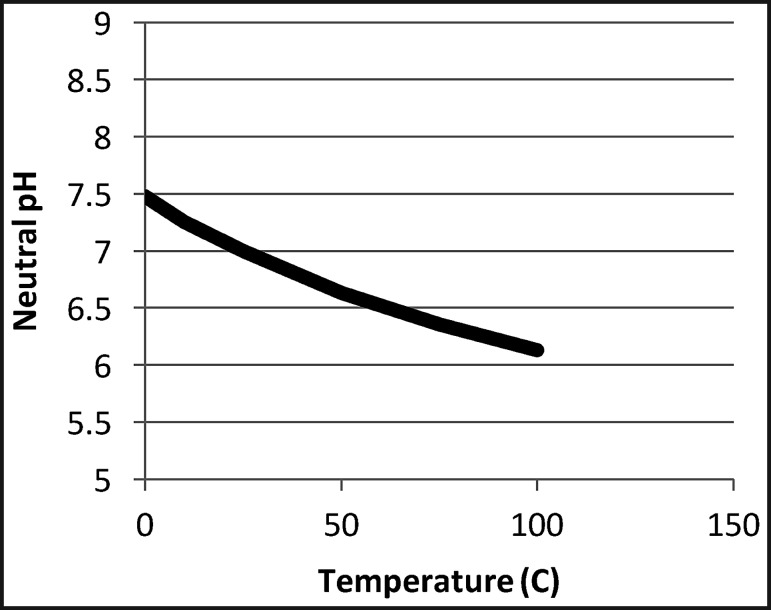


A diagenetically precipitated silica matrix that rapidly sealed and lithified all components and signatures in the JC, leading to final observed precipitation, was finally preserved by rapid diagenetic sealing and lithification by a precipitated silica matrix (van den Boorn *et al.,* 2007; Sugitani *et al.,* 2015; Westall *et al.,* 2015a; Tartèse *et al.,* 2017). Rapid diagenetic sealing prevented later elemental composition changes.

#### 2.2.3. Geochemical evidence

**FIG. 8.**
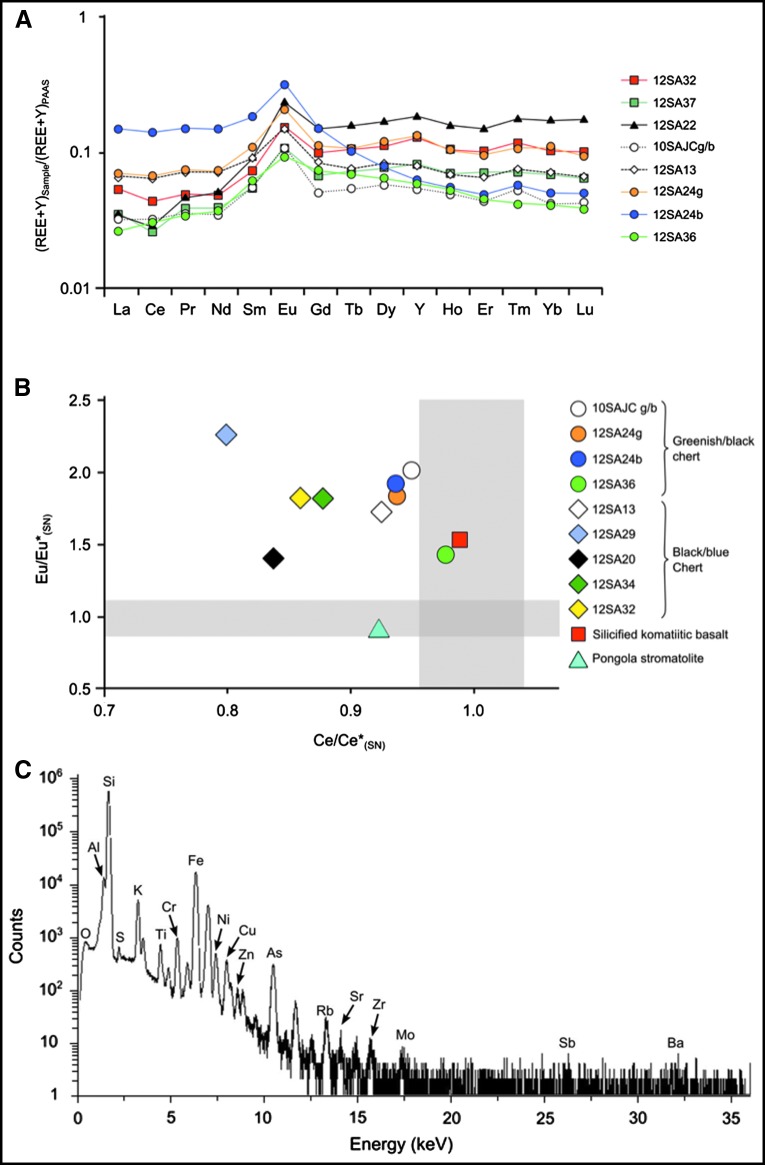
Geochemical analyses demonstrating the bulk influence of hydrothermal fluids on the JC sediments (from Hubert, 2015). (**A**) Shale-normalized (PAAS, McLennan, 1989) REE+Y patterns of several samples from the JC. The positive Eu anomaly is indicative of a hydrothermal influence (Danielson *et al.,* 1992; Derry and Jacobsen, 1999). (**B**) Plot of Eu and Ce anomalies (shale-normalized; PAAS, after McLennan, 1989). Eu/Eu*: Eu/((Sm*05) + (Gd*05)) and Ce/Ce*: Ce/((La*05) + (Pr*05)). An Eu/Eu* value (related to the Eu anomaly) of >1 indicates a hydrothermal signature (Danielson *et al.,* 1992; Derry and Jacobsen, 1999), while Ce/Ce* (related to a La anomaly) indicates a strong marine signal where <1. The results from two additional samples, silicified carbonates of the 2.9 Ga silicified stromatolites of the Pongola Supergroup, South Africa, and a silicified komatiitic basalt from Josefsdal are given for comparison. The Pongola stromatolite displays a marine signature (Ce/Ce* <1 plus La anomaly) but no hydrothermal signature, while the silicified basalt exhibits a hydrothermal signal (Eu/Eu* of >1 plus Eu anomaly) without marine influence. Josefsdal sediment samples show mixed signatures indicating fluids influenced by both marine waters and hydrothermal activity. (**C**) PIXE spectrum acquired for 8 h with a 2 μm diameter proton beam from a hydrothermal chert vug in highly silicified Facies D sediments (after Westall *et al.,* 2015a) showing the presence of a number of hydrothermally transported elements, including Fe, Ni, Cu, Zn, As, and Ba.

**FIG. 9.**
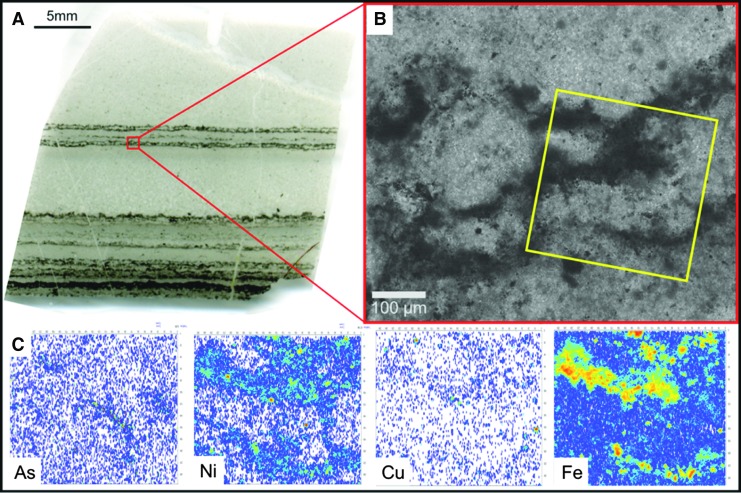
Hydrothermal element scavenging by altered volcanic particles. (**A**) Thin section of sedimented volcanogenic particles showing dark layers comprising concentrations of volcanic particles (including spherules), as well as traces of carbon and microcrystalline pyrite (Facies D, after Westall *et al.,* 2015a). (**B**) Optical micrograph of the volcanic particles in a black layer in the JC sediments. Yellow box denotes the area of the regions in (C). (**C**) PIXE elemental maps (beam size: 2 μm; map size: 300 × 300 μm; resolution: 256 × 256 pixels; 3.5 h acquisition) showing the concentration of Fe, As, Ni, and Cu, trace elements of hydrothermal genesis scavenged by the altered volcanic particles.

Bulk geochemical analyses in Fig. 8A show shale-normalized (PAAS; McLennan, 1989) REE+Y patterns of several samples from the JC (Fig. 8), documenting a positive Eu anomaly (Figs. 8A–8B), indicative of hydrothermal influence (Danielson *et al.,* 1992; Derry and Jacobsen, 1999). In Fig. 8B, the plot of Eu and Ce ratios further refines the results by showing that, while the hydrothermal influence is very strong, there is also an admixture of seawater, with different samples showing different degrees of mixing (Danielson *et al.,* 1992; Derry and Jacobsen, 1999). Finally, the particle-induced X-ray emission (PIXE) spectrum in Fig. 8C, from a strongly hydrothermally influenced JC sample (Facies C, Westall *et al.,* 2015a), documents the presence of hydrothermally transported elements, including Fe, Ni, Cu, Zn, As, and Ba. The scavenging of this hydrothermal signature by carbonaceous material is demonstrated in Fig. 9. Furthermore, the incorporation of large amounts of Ni into early diagenetic pyrites of the JC, from which up to 3 wt % may have been provided by hydrothermal circulation, additionally suggests the early diagenetic influence of hydrothermal fluids (Hubert, 2015).

## 3. Discussion

### 3.1. A hydrothermal environment

The above observations relating to the physical, mineralogical, and geochemical evidence for hydrothermal fluid influence on the JC sediments clearly show that hydrothermal fluids permeated the sediments during their deposition, mixed to varying degrees with the ambient seawater, formed chemical deposits, and continued interaction with the sediments from the early to late diagenetic phase.

#### 3.1.1. Silica gels

A significant observation of this study relates to the importance of silica gels filling in pore spaces between the volcanic particles, as well as precipitating as chemical sediments. Silica gels are porous, granular deposits containing fluids inside nanosized pores. These silica gels fill and thereby preserve the pores in the sedimentary deposits on timescales related to both silica saturation in the pore fluids and pH. Further, as shown above, pH values in the sediment pore spaces of volcanic particles that are intermixed with slightly acidic seawater will initially rapidly increase (Fig. 4A). The increase in pH corresponds to alteration and devitrification of the volcanic particles, thus adding to silica saturation of the pore fluids. The decrease in pH would have influenced precipitation of the silica as silica gel. Such a mineral phase in direct contact with the reactive mineral surfaces of volcanic particles would have reduced water activity in the pore spaces, thus protecting organic molecules from hydrolysis, while at the same time limiting the range of potential chemical reactions of the organic molecules. Indeed, gels could be considered as an example of inorganic cells, protecting organic molecules, while allowing energy dissipation and molecular mobility.

#### 3.1.2. Mixing

We have documented mixing of hydrothermal fluids (with varying amounts of admixed seawater) with the reactive volcanic sediments, the latter characterized by particle surfaces exhibiting microscale morphological variability accompanied by microscale mineralogical variability and associated elemental distribution (Figs. 3–4). The mixing of carbon with the sediments and the hydrothermal fluids is important within the context of silica gels. As noted above, carbon in the JC sediments is of biogenic and abiogenic origin, but for this discussion its origin is not relevant, since its usefulness is (i) to show the presence of carbon in ancient sediments (in the prebiotic world, the carbon would have been of purely abiotic origin) and (ii) to document mixing of carbonaceous sediment with, and by, hydrothermal fluid as documented in Figs. 2 and 6.

### 3.2. Physicochemical characteristics of a hydrothermal sedimentary environment

Table 5 summarizes the characteristics of the kind of hydrothermal sedimentary environment epitomized by JC sediments. Temperatures within the sediments deposited at the interface between hot lavas and seawater, and intruded and permeated by hydrothermal fluids, would have been warm to hot. Various studies to quantify temperatures of Early Archean cherts show temperatures ranging from low, ∼30°C (Hren *et al.,* 2009), to relatively high, ∼60°C (Tartèse *et al.,* 2017). van den Boorn *et al.* (2007, 2010) showed that temperature ranges in a similar sequence of sediments (Kitty's Gap Chert in the Pilbara; *cf.* Westall *et al.,* 2006b) reflected fluctuating input of hydrothermal fluids and admixture with seawater. Values of pH would have varied from alkaline to acidic, depending upon input of hydrothermal fluids mixing with seawater and changing pH in pore waters during alteration of the volcanic particles. We have seen that the fluid dynamical situation in the sediment was variable, ranging from very quiescent conditions in which hydrothermal fluids simply permeated the sediments to continued forceful and disruptive intrusions that mixed all components together on longer timescales. The ionic strength of the fluids in these sediments would have been high, with a plenitude of ions being sourced from seawater, hydrothermal fluids, and the altered volcanic particles. Energy sources would have been provided from heat from the hydrothermal vents and fluids, as well as by ubiquitous, exothermic redox reactions occurring during aqueous alteration of the surfaces of the volcanic particles, the mineralogy of which consisted predominantly of volcanic glass, pyroxene, plagioclase, olivine, and their alteration minerals, together with phyllosilicates and anatase (tourmaline occurs elsewhere in similar Onverwacht Group sediments). Secondary minerals included Fe carbonate, pyrite, and barite. A meteoritic/micrometeoritic component also would be expected (*cf.* Lowe *et al.,* 2003; Maurette and Brack, 2006), bearing a wide range of minerals depending upon their composition (stony, iron, carbonaceous chondrite, etc.), including common pyroxene, olivine, feldspar, Fe-Ni alloys, oxides (chromite, ilmenite), and many rarer secondary species including the P-bearing mineral schreibersite (Rubin, 1997). Organic components were imported via hydrothermal fluids (*cf.* Fig. 7B) and probably also in the carbonaceous chondritic portion of the meteoritic input. Sediment porosity varied depending on grain size and depositional processes, but permeability was assured even in the finer-grained sediments, as testified by the formation of hydrothermal siderite particles within these sediments. Deposited in water depths ranging from wave base to shore face (beach), the sediments offered some to little protection from UV radiation, depending upon water depth and depth below the sediment surface, in the case of shallow-water sediments (UV attenuation depth in different sediment types ranges from 4 to 21 mm; Garcia-Pichel and Bebout, 1996).

**Table 5.** Environments Available for the Emergence of Life

*System*	*Prebiotic chemistry*		*Emergence of life*	
*Properties*	*Advantages*	*Disadvantages*	*Advantages*	*Disadvantages*
**Submarine hydrothermally influenced sediments**				
Temperatures ∼50 to >100°C	Facilitates molecular interactions		Temperatures acceptable for protocellular activity	
Fluid dynamics	Low to high	High dynamics will disrupt molecular bonds	Diffusion of nutrients	
pH—alkaline-acid	Favors prebiotic processes		Any pH for protocells	
Ionic strength	Variable		Salts necessary for protocells	
Energy sources	Heat, redox reactions		Small organics (from Fischer-Tropsch and ultramafic fluid inclusions); redox reactions of reactive minerals; gradients (pH, temperature…)	
Mineralogy, *e.g.,* sulfides and various mafic/ultramafic minerals and their alteration products	Reactive surfaces favor molecule-organic interactions (Table 1)		Energy from redox reactions at mineral surfaces	
Element availability	CHNOPS		CHNOPS, Cu, Fe, Ni, Zn…	
Organics (from hydrothermal fluids, volatile and degraded ET organics)	Volatiles, small organic molecules, components of macromolecular building blocks of life		Presence of nutrients (small organics, CH_4_)	
Porous sediments	Concentration of prebiotic components, compartmentalization		Protected environment for protocells	
Silica hydrogels	Very common, compartmentalization, confinement		Hydrogels as protocells, compartmentalization and autoreplicative system	
Protection from external environment	UV protection, disruption caused by impacts		Protected environment—from UV, etc.	
Distribution of products	Mixing of pore water and sediments with seawater, current transport		Mixing of pore water and sediments with seawater, current transport	

An added advantage of the kind of 3-D environment envisaged in the sediment/silica gel/hydrothermal fluid mixture is the continual access to essential ingredients for prebiotic synthesis, a situation that does not exist in a 2-D environment, for instance.

Table 2 summarizes the physicochemical characteristics of environments that would favor prebiotic reactions. The characteristics of the hydrothermal volcanic sediments described here compare well with the conditions favorable for prebiotic reactions.

### 3.3. Relevance of Paleoarchean sediments for the Hadean prebiotic environment

How relevant would the Paleoarchean sediments of the JC be for a Hadean origin-of-life scenario? Would such a hydrothermal volcanic sedimentary environment have existed in the Hadean? Despite the lack of Hadean rock record, there is some remnant geochemical record from buried Hadean cratons; moreover, we have knowledge of the nature of the Paleoarchean crust. Thus, given this information and our understanding of planetary evolution, it is legitimate to envisage, at the local scale, the formation of thin layers of volcanic sediments on basaltic crust at various water depths, but especially on submerged continental crust and rare exposed land areas. Heat flow from the crust would have ensured vigorous hydrothermal circulation. At this scale and from this point of view, the JC sediments can be considered as relevant analogues for Hadean sediments and thus a suitable environment for prebiotic processes leading to the emergence of life. Prebiotic reactions could have taken place in hydrothermally influenced volcanic sediments wherever such environments occurred on the Hadean Earth and may, indeed, have been widespread.

### 3.4. Comparison of hydrothermal volcanic sediments with other origin-of-life scenarios

In any assessment of a putative geological setting for the origin of life, the environment in question must meet the three critical criteria of origination, complexification, and plausibility. These three parameters are defined as follows:

• Origination—the ability of the environment to provide the molecular and mineral components that co-facilitate prebiotic reactions;
• Complexification—the ability of the environment to sustain conditions conducive to both continued directional reactions and the overall diversification of the molecular complement of the system;
• Plausibility—the relevance of the environment to, and supposed survival on, the Hadean Earth, based upon available geological evidence.

**FIG. 10.**
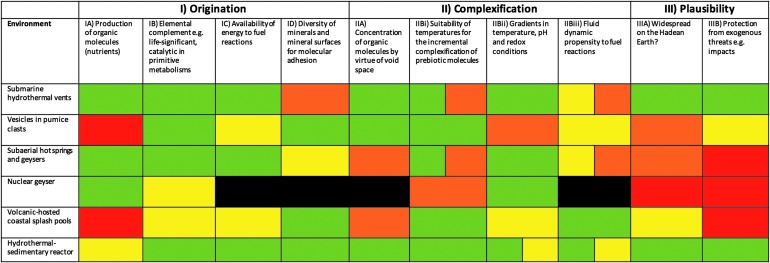

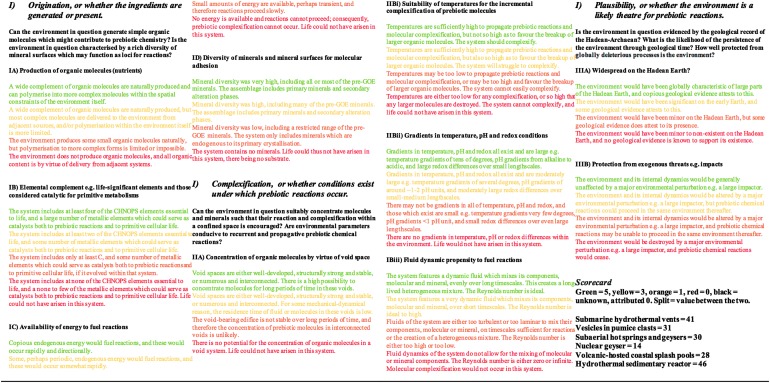
“Traffic Light— diagram comparing the potentials of the proposed environments for the origin of life. The conditions necessary for prebiotic complexification leading to the origin of life are split into origination (the ability of the environment to provide the molecular and mineral components that co-facilitate prebiotic reactions), complexification (the ability of the environment to sustain conditions conducive to both continued directional reactions and the overall diversification of the molecular complement of the system), and plausibility (the relevance of the environment to, and supposed survival on, the Hadean Earth, based upon available geological evidence). The text below the diagram outlines the rationale for the assignment of the color code. See main text for further interpretation of this diagram.

The multifaceted aspects that comprise these criteria for each environment described above (Tables 3a–3d for hydrothermal vents, pumice rafts, subaerial geysers/nuclear hot spots, and volcanic-hosted coastal splash pools; Table 5 for hydrothermal volcanic sediments) are examined in Fig. 10. This modified “Traffic Light” diagram separates each criterion into five color-coded enumerations: green (entirely meets the criterion), yellow (generally meets the criterion with minor caveats), orange (may or may not meet the criterion due to major caveats), red (wholly fails to meet the criterion), and black (unknown or unconstrained).

We consider that any environment that has two “red lights” in the plausibility columns should be considered no further, since its existence is either implausible based on the geological record or evidentially unsupported by the ancient geological record. Five of the six environments are thus judged plausible, although subaerial hot springs (and indeed the continents upon which they would have been situated; *cf.* Kamber, 2015) were likely minor and barely pass this first test. Additionally, more than one “red light” in the origination columns is deemed a major setback in the ability of this system to facilitate prebiotic chemistry. Therefore, for pumice clasts and volcanic-hosted splash pools, their inability to generate their own organic complement makes necessary their interaction with another environment. Consequently, while these environments have numerous strengths in the complexification stage, their effectiveness in the origination stage is poor.

The ideal setting for the origins of life would have strengths in origination and complexification, that is, its potential for prebiotic chemistry, while having no weaknesses in plausibility. The terrestrial nuclear geyser hypothesis is clearly implausible, while subaerial hot spring systems were definitely minor and short-lived. Volcanic-hosted terrestrial splash pools and pumice clasts suffer from an inability to generate their own prebiotic complement and are susceptible to disruption by catastrophic geological events. Only two environments pass the criteria set with mostly “green lights”: submarine hydrothermal systems and the hydrothermal-sedimentary reactor. Of the two, based on our observations described from Hadean analog sediments herein, the hydrothermal-sedimentary reactor has two key advantages in complexification that make it a superior setting for prebiotic chemistry:

• The more clement temperatures in this “distal” hydrothermal system are more conducive to directional prebiotic chemistry complexification reactions without the destruction of longer polymers.
• The fluid dynamics of the system is variable, leading to both mixing and quiescent conditions. This would result in higher probabilities of multiple reactions over a longer timescale and, broadly, allow more time for the totality of prebiotic complexification to occur.

A further advantage of the hydrothermal volcanic sediment scenario is the ubiquitous presence of silica gels, the advantages of which for prebiotic chemistry have been enumerated above. However, although never addressed in relation to the other environments evoked for the origin of life, silica gel could also be associated with hydrothermal vents themselves during the waning stages of their activity and also with subaerial geysers, precipitating during evaporation. Moreover, silica-saturated seawater could have precipitated in coastal splash pools also during evaporation. Finally, the porous nature of pumice rafts and their specific location at the air/water interface suggest that silica gel might have been precipitated during evaporation. Thus, to differing degrees, all the proposed environments could have had the additional advantage of silica gels.

Alkaline submarine hydrothermal vents were certainly an important part of the most plausible geological setting for the origins of life on Earth (Russell and Hall, 1997; Martin and Russell, 2003; Russell, 2007; though, for an alternative viewpoint, see Jackson, 2016). Here, we invoke aspects of this system in a more mineralogically diverse setting, that is, within hydrothermal volcanic sediments, and with more moderate dynamic conditions, as an alternative and likely ubiquitous setting for prebiotic molecular complexification leading to the appearance of life.

## 4. Summary and Conclusions

**FIG. 11.**
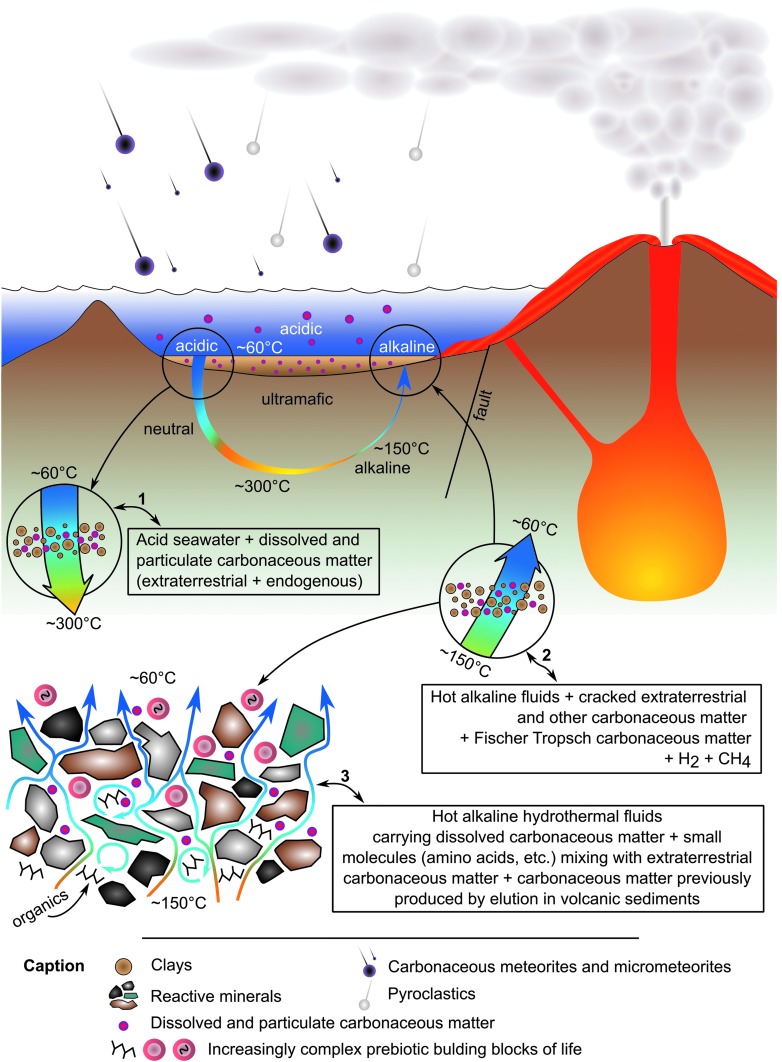
Schematic synthesis of the proposed Hadean, hydrothermal-sedimentary micro-reactor environment for complexification of prebiotic chemistry. Slightly acidic seawater entraining dissolved and particulate carbonaceous matter of diverse origins permeates through ultramafic/mafic sediments into the crust (insert 1), altering the ultramafic rocks and becoming more alkaline during these reactions. Light-weight carbon molecules and gases (*e.g.,* H_2_, CH_4_) formed by Fischer-Tropsch-type processes (Shock *et al.,* 2002), as well as molecules from ultramafic fluid inclusions (Van Kranendonk *et al.,* 2015), were convected into reactive porous sediments at the bottom of the sea (the sediment-water interface, insert 2), where a temperature and pH disequilibrium (insert 3) with the overlying acidic seawater existed. Convection of warm, carbon-bearing hydrothermal fluids allowed prebiotic molecules to concentrate and self-assemble in pore spaces and on the surfaces of chemically reactive minerals, resulting in the formation of increasingly complex molecules.

Our observations on Paleoarchean sediments judged analogous to those in similar Hadean environments document an environment consisting of reactive volcanogenic sediments deposited at the interface between volcanic lithosphere and the hydrosphere, a seawater-sediment interface strongly influenced by hydrothermal fluids and rapid alteration of mafic-ultramafic minerals, and therefore characterized by temperature and pH disequilibria (Westall *et al.,* 2015b; Westall, 2016; Fig. 11). Alkaline and highly reduced (H_2_-rich) hydrothermal fluid effluent, as well as alkaline fluids resulting from the alteration of volcanic grains, would have intermixed with the relatively more acidic (CO_2_-rich) ocean water, creating a gradient in redox conditions (Martin *et al.,* 2008) within the sediments and at the sediment-water interface. Temperature disequilibria would have resulted from the variable pulsing of hydrothermal fluids within the sediments and mechanical mixing with the overlying seawater. The natural heterogeneity of the sedimentary materials described here (mineralogy, chemistry, porous texture, particle morphology, pore-fluid pH, and temperature) provided for a plurality of surface-solution interactions akin to a contiguous, flow-through chemical reactor. The sediments, together with silica gel, would create a network of pore spaces, each with a local microenvironment conducive to producing a range of prebiotic organic molecules. Within the pores, interaction of water with the functional groups (silanol) of the silica gel and mineral grains would have limited the ability of water molecules to hydrolyze the organic molecules attached to the minerals, thus facilitating the formation of building blocks of peptides and nucleotides, for example. These gels, associated with minerals in the sediments, could have acted as a diffusional barrier to achieve the required retention time for complex chemistry to emerge (*cf.* Trevors and Pollack, 2005). In this setting, heat-driven convection would have been fundamental for transporting reactants to multiple reaction sites (Simoncini *et al.,* 2011). The hydrothermal pathways through sedimentary systems could have distributed soluble compounds leading to their distal accumulation.

From the perspective of prebiotic chemistry, sedimentary systems on the primitive Earth offered possibilities for the processing of raw organic matter provided by serpentinizing systems, liberated from ultramafic fluid inclusions, and delivered by meteoritic infall. While serpentinizing systems are highly attractive as a source of both the reducing potential and carbon required to build a molecular inventory (Baross and Hoffman, 1985; Russell and Hall, 1997; Martin *et al.,* 2008; Sleep *et al.,* 2011; though see McCollom and Donaldson, 2016, for an alternate view), to date little attention has been given to the chemical modification of extraterrestrial carbon in early hydrothermal-based theories for life's origins (Baross and Hoffman, 1985; Russell and Hall, 1997; Martin and Russell, 2007; Martin *et al.,* 2008; Simoncini *et al.,* 2011). As noted above, meteorite and micrometeorite analyses strongly support early, massive, and widespread delivery of extraterrestrial organics, corresponding to as much as 10% of the oxidized carbon remaining in the modern biomass (Sephton, 2002) and additionally providing minerals containing potentially reactive metals (Maurette, 2006; Pizzarello and Shock, 2010). About 75% of the carbonaceous matter distributed within carbonaceous chondrites is insoluble macromolecular material (Pizzarello and Shock, 2010), which would have concentrated in the sediments. Subsequently, reaction with hot fluids would have contributed to the breakdown of recalcitrant organic matter and its subsequent availability for prebiotic chemistry on reactive mineral surfaces within the sediments.

Thus, porous, reactive sediments on a hydrothermally active Hadean Earth provided a UV-protected environment at a critical chemical, thermal, and dynamical disequilibrium interface (Table 1) and could have hosted globally distributed, miniature chemical reactors in the form of sedimentary pore space, as summarized in Fig. 11.

## Appendix A. Materials and Methods

Our study is based on extensive field and microscopic examination of silicified volcanic sediments ranging from 3.46 to 3.33 billion years in age, from the Barberton Greenstone Belt in South Africa and the Pilbara in Australia. More specifically for this investigation, we have made a detailed, complementary microscopic, mineralogical, and geochemical study of a suite of samples from the JC (3.33 Ga, Westall *et al.,* 2015a) in the Barberton Greenstone Belt (Fig. 1). Samples were collected during field campaigns between 1999 and 2014.

Optical microscopy was conducted on 30–60 μm polished thin sections using an Olympus BX51 microscope at CNRS-CBM (Orléans). Scanning electron microscopy, in backscatter mode, of the surfaces of uncoated, polished thin sections was conducted at CNRS-ISTO (Orléans), with a Hitachi TM3000 Tabletop SEM equipped with a Swift ED3000 silicon drift detector operating at 15 kV. Elemental maps were acquired with a resolution of 512 × 384 pixels for 60 min using a line length of 75.4 μm (magnification × 1500). Raman analyses were undertaken at CNRS-CBM (Orléans) on polished thin sections using a WITec Alpha500 RA Raman spectrometer following the method described in Westall *et al.* (2015a). Whole rock analyses were conducted utilizing laser ablation ICP-MS and ICP-OES at the University of Cardiff. The REE concentrations were normalized to PAAS shale (McLennan, 1989). *In situ* elemental mapping was undertaken on 60–80 μm thick rock sections by PIXE at the AIFIRA facility (Applications Interdisciplinaires des Faiseaux d'Ions en Région Aquitaine), CENBG (Bordeaux-Gradignan). Acquisitions were made with 3 MeV proton microbeam (1 μm diameter) using two Si(Li) detectors equipped with (1) Al 100 μm thick Mylar “funny” filter with a 2 mm hole and a 50 μm thick kapton filter used to eliminate the Si signal; (2) 100 μm thick Al “funny” filter with a 1 mm hole. Beam intensity is kept low enough to have less than 10% dead time on detectors.

The composition of Hadean seawater (Table 4) was calculated by PHREEQC (Parkhurst and Appelo, 1999) with the llnl (Lawrence Livermore National Laboratory) thermodynamic database (Johnson *et al.,* 2000; Blasco *et al.,* 2017). The initial suite of ultramafic/mafic minerals represented the komatiites and basalts present during the Hadean (Arndt, 1994; Hazen *et al.,* 2008). These minerals were fosterite, bytownite, diopside, and ilmenite (Table 2). Two models were tested: (i) an initial solution of hot (50–100°C), CO_2_-rich (PCO_2_ 1–10 atm) solution, equilibrated with the mineral suite by dissolution only without additional gas equilibration, representing advection of acidic seawater through the igneous rocks without additional equilibration with the atmosphere; and (ii) simultaneous equilibration of the minerals with atmospheric PCO_2_, representing continuous connection of hydrothermal fluids with acidic seawater (Table 3).

## Notes

^1^ Note that information pertaining to materials and methods is in the appendix.

## Abbreviations Used

JC: Josefsdal Chert
PIXE: particle-induced X-ray emission
